# Sudden cardiac arrest: Limitations in risk-stratification and treatment, and the potential for digital technologies and artificial intelligence to improve prediction and outcomes

**DOI:** 10.1016/j.pcad.2025.06.005

**Published:** 2025-06-18

**Authors:** Sudarshan Srivats, Fawzi Zghyer, Zaid Shahrori, Christine Albert, Sana M. Al-Khatib, Sumeet Chugh, Susan P. Etheridge, Zachary D. Goldberger, Rakesh Gopinathannair, Dhanunjaya Lakkireddy, Daniel P. Morin, Marco V. Perez, Markus Rottmann, Jacob E. Sunshine, Paul J. Wang, Mina K. Chung

**Affiliations:** a The Department of Hospital Medicine, Catholic Medical Center, Manchester, NH, USA; b The Department of Cardiovascular Medicine, Heart, Vascular and Thoracic Institute, Cleveland Clinic, Cleveland, OH, USA; c The Department of Internal Medicine, University Hospitals Cleveland Medical Center, Case Western Reserve University, Cleveland, OH, USA; d Department of Cardiology, Smidt Heart Institute, Cedar-Sinai Medical Center, Los Angeles, CA, USA; e Division of Cardiology and Duke Clinical Research Institute, Duke University Medical Center, Durham, NC, USA; f Center for Cardiac Arrest Prevention, Department of Cardiology, Smidt Heart Institute, and Division of Artificial Intelligence in Medicine, Department of Medicine, Cedars-Sinai Medical Center, Los Angeles, CA, USA; g Pediatric Cardiology, St. Luke’s Children’s Hospital, Boise, ID, USA; h Division of Pediatric Cardiology, Department of Pediatrics, Lucile Packard Children’s Hospital Stanford, Palo Alto, CA, USA; i Department of Medicine, Division of Cardiovascular Medicine, University of Wisconsin-Madison, School of Medicine and Public Health, Madison, WI, USA; j Department of Cardiology/Electrophysiology, Kansas City Heart Rhythm Institute, Overland Park, KS, USA; k Department of Medicine, Division of Cardiology, University of California San Francisco, San Francisco, CA, USA; l Division of Cardiovascular Medicine, Stanford University School of Medicine, Stanford, CA, USA; m Feinberg Cardiovascular and Renal Research Institute, Feinberg School of Medicine, Northwestern University, Chicago, IL, USA; n Department of Medicine, Section of Cardiology, University of Chicago, Chicago, IL, USA; o Department of Medicine, Department of Anesthesiology and Pain Medicine, University of Washington, and Paul G Allen School of Computer Science and Engineering, Seattle, WA, USA

**Keywords:** Sudden cardiac death (SCD), Sudden cardiac arrest (SCA), Risk stratification, Artificial intelligence (AI), Wearable devices

## Abstract

Sudden cardiac death (SCD) remains a pervasive public health challenge, accounting for a significant proportion of cardiac and all-cause mortality worldwide. Despite notable advancements in cardiovascular therapies and reductions in overall cardiac mortality, survival following sudden cardiac arrest (SCA) remains dismally low, and prediction strategies remain inadequate. This comprehensive review examines the current landscape of SCD etiologies and the latest guidelines for primary and secondary prevention of SCD with implantable cardioverter defibrillators (ICDs). Particular attention is given to the limitations of left ventricular ejection fraction (LVEF) as the primary tool for risk stratification, given its low sensitivity, specificity, and limited applicability to the broader population in which most SCDs occur. Emerging risk scores and machine learning (ML) driven prediction models have begun to efficiently integrate clinical, electrical, imaging, genetic and laboratory parameters to improve SCD risk stratification. This review highlights examples of such artificial intelligence (AI) prediction models and discusses their potential role in the near-term and long-term prediction of SCD in both in-hospital and out-of-hospital settings, while emphasizing the need for external validation of such models. The review also discusses critical system-level gaps in the chain of survival from cardiac arrest, particularly the need for automated emergency medical services (EMS) activation, community responder engagement, high-quality cardiopulmonary resuscitation (CPR) and improved access to defibrillation. It explores the role of digital technologies such as wearable sensors, smartwatches, smartphone applications and implantable devices in improving real-time SCA detection and enhancing early aspects of the chain of survival from cardiac arrest. Finally, the review calls for a multidisciplinary, multi-sectoral approach including regulatory, technological, and public health stakeholders to bridge gaps in SCD prevention, detection, and response.

## Introduction

**Fig. 1.**
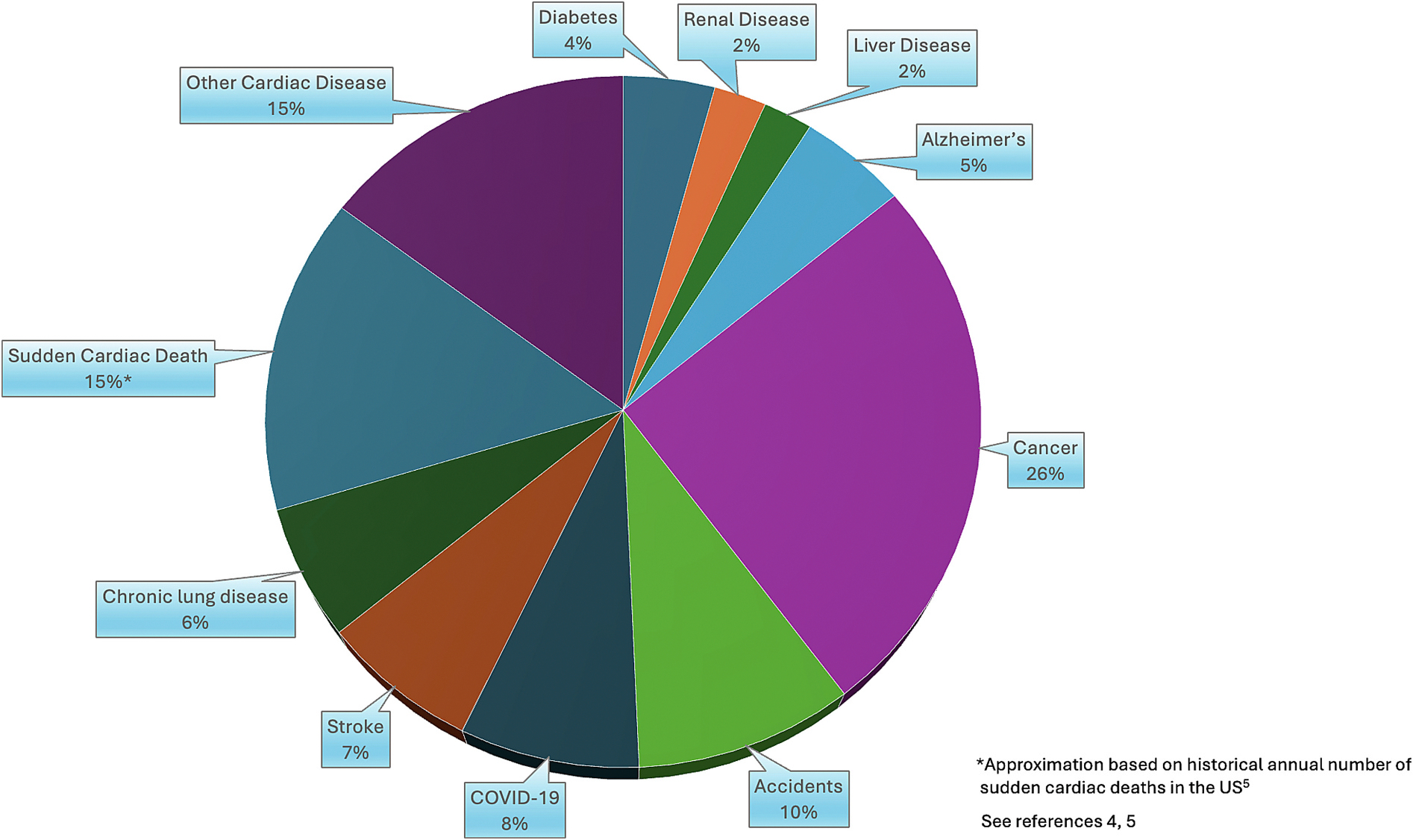
Annual Mortality in the United States by Etiology - From 2022 CDC Data^4^.

Sudden cardiac death (SCD) is often defined as an unexpected death without obvious noncardiac cause that occurs in association with a witnessed rapid collapse or within 1 h of the onset of symptoms.^1^ Sudden cardiac arrest (SCA) is defined similarly, but also includes survivors of the arrest. With over 356,000 SCAs in the US and over 3–4 million SCD per year globally, SCD remains a pervasive public health burden, accounting for around half of cardiac deaths and 15–20 % of all-cause mortality^2,3^ (Fig. 1).^4,5^ Despite major advances in cardiovascular medicine that have decreased overall cardiac deaths, mortality due to SCA remains high, with overall survival rates around 10 %.^6,7^

A major challenge is that in almost half of SCD cases, SCD is the initial presentation, with limited or no prior recognized symptoms or warning signs for underlying cardiovascular disease (CVD).^8^ For some, symptoms or signs may occur shortly before the arrest, but do not elicit a response or without enough time to get help. In most cases, however, SCD is a manifestation of previously undetected CVD.^9,10^ Therefore, there is a critical need to formulate better screening strategies to identify subclinical CVD, risk factors for coronary artery disease (CAD) in the general population, and other indicators of impending arrhythmic events. A second challenge is the need for better prediction models for SCD in those with known CVD. SCD is a complex event with multiple interacting risk factors, including chronic diseases and acute triggers. Our most established risk stratification tool, the left ventricular ejection fraction (LVEF), lacks both sensitivity and specificity.^9,11^ A third main challenge is the poor outcomes following SCA which is dependent on multiple aspects of resuscitation. This review identifies the current gaps in SCD prediction models and examines how novel approaches that utilize digital sensors, other health technologies, and artificial intelligence (AI) and machine learning (ML), could build better prediction models and improve SCA outcomes.

## Etiologies of SCD

Based on autopsies of SCD victims and evaluation of SCA survivors, CAD is the most common etiology for SCD in adults over age 35, accounting for about 70 % of all SCD.^12^ The prevalence of CAD varies across sex and races. In autopsy and survivor series, the prevalence of CAD among those suffering SCA is 45–50 % in women, versus 80–90 % in men, and may be lower among African-Americans with SCD compared to similarly afflicted Caucasian individuals (47 % vs 63 %).^12^

Cardiomyopathies are the second most common etiology for SCD in the adult, accounting for about 10–15 % of overall SCDs.^13^ These include dilated cardiomyopathy (DCM), hypertrophic cardiomyopathy (HCM), arrhythmogenic cardiomyopathy (ACM) and infiltrative diseases (e.g., sarcoidosis, amyloidosis).^14,15^ The remaining common causes of SCD include inherited channelopathies (representing 1–2 % in Western countries, and up to 10 % in Japan) and valvular heart disease (Table 1). The prevalence of these non-ischemic etiologies for SCD is higher in younger individuals (<35 years).^16^ Lastly, in adults and children, a large proportion of SCDs remain unexplained after autopsy. Some studies have estimated this proportion to be as high as 30 to 40 % in persons aged 1 to 49 years.^17,18^

## SCA in various risk populations

When SCD risk is analyzed by subgroups of the adult population, as one would expect, the highest risk clinical groups include patients with prior coronary events, reduced LVEF, history of heart failure (HF), and survivors of SCA, who have much higher incidences of SCD compared with lower risk clinical subgroups and the overall adult population. For example the overall incidence of SCD in the US adult population is 0.1–0.2 % per year but the incidence of SCD in survivors of SCA is >20 %.^19^ However, in terms of the absolute number of annual SCD events, the higher risk clinical subgroups have low numbers of annual SCD events compared with the lower risk groups and the general adult population.^19^ The risk profiles of various population subgroups and current guidelines for primary and secondary prevention of SCD are discussed below.

### Survivors of SCA

Survivors of SCA or sustained or hemodynamically significant ventricular tachycardia (VT) or ventricular fibrillation (VF) are known to be at very high risk for recurrence. These patients are candidates for implantable cardioverter defibrillators (ICDs). Randomized clinical trials that studied the survival benefits of ICDs for secondary prevention of SCD in patients resuscitated from cardiac arrest and near fatal arrhythmias include the Antiarrhythmics versus Implantable Defibrillators (AVID) trial^20^, Cardiac Arrest Study Hamburg (CASH)^21^, and Canadian Implantable Defibrillator Study (CIDS)^22^ trials. Results from the AVID trial led to class I recommendations for ICD implantation in these patients. These trials are summarized in Table 2.

### SCA risk in children and adults with congenital heart disease

The annual pediatric incidence of SCD can vary depending on age-specific groups but overall, it is estimated to be around 7.5 per 100,000 children.^25^ When considering SCD in the young, one must consider those with and without congenital heart disease (CHD). The incidence of CHD is 8–9 per 1000 live births, and while rare, it can lead to SCD during childhood and beyond, often a result of residual hemodynamic burdens, scars from previous surgeries, and/or damage to critical structures during surgery. Higher-risk substrates include tetralogy of Fallot, transposition of the great arteries, cyanotic heart disease, Ebstein anomaly, and those with a Fontan circulation.^26^ High-quality CPR is essential to improve outcomes, but pulmonary hypertension and CHD complexity are associated with a lower likelihood of successful resuscitation. Risk stratification scores to identify the appropriate population for primary prevention ICD implantation in the adult CHD (ACHD) population have been proposed and validated.^27^ The PREVENTION-ACHD risk score was based on clinical factors: coronary artery disease, NYHA Class, supraventricular tachycardia (SVT), systemic ventricular EF <40 % and subpulmonary ventricular function EF <40 % (if a subpulmonary ventricle is present), QRS duration >120 ms, and QT dispersion >70 ms. The score identified patients with ACHD at high SCD risk with a hazard ratio (HR) for SCD or VT/VF of 11.9 in high-risk patients compared with low-risk patients and a C-statistic of 0.75.^27^ However, this risk score has not been validated in children with CHD.

### SCD risk in the general adult and child population

Unfortunately, predicting SCD in the general population without a history of heart disease is challenging. Screening tests such as electrocardiograms (ECG) could lead to increased diagnoses of underlying CVD in otherwise asymptomatic individuals, which in theory may lead to the identification of members of the general population who are at risk for SCD. However, the United States Preventive Services Task Force has determined that there is insufficient evidence to recommend routine ECG screening for CVD in the general population.^28^ Specifically, the current evidence does not adequately assess the balance of benefits and harms associated with using resting or exercise ECGs to screen for CVD and prevent related complications.^28^ A high rate of false positive tests with such screening strategies can cause unnecessary patient distress and overburden the healthcare system. Therefore, there is no screening strategy currently recommended for SCD risk stratification in the general population. Nevertheless, identifying and modifying potentially modifiable risk factors for CVD may reduce SCD risk.

Although the presence of structural or electrical heart disease increases the risk of SCD, the majority of SCD occurs in individuals who have not had CVD diagnosed before the event.^8,10,29^ Reducing SCD risk in this segment of the population could involve risk factor and lifestyle modification. Some factors, such as hypertension (HTN), diabetes mellitus, hypercholesterolemia, obesity, and smoking, may elevate SCD risk because they increase the risk for CAD.^29–33^

In children there are multiple conditions that predispose to SCD that could be identified pre-mortem. These differ from the causes in the adult population, with a greater incidence of inherited diseases such as long QT syndrome (LQTS), hypertrophic cardiomyopathy (HCM), catecholaminergic polymorphic ventricular tachycardia (CPVT), Wolff Parkinson White syndrome (WPW), and coronary artery anomalies. The incidence of genetic conditions that pose a risk of SCD is as common as 1 in 500 for HCM and 1 in 2000 for CPVT, Brugada syndrome (BrS) and LQTS. One must consider that these are relatively common conditions that must have some overlap. It is important to note that published series of young SCD (< age 35 years) have found that the most common group is those with autopsy negative sudden death.^18^ This includes CPVT, LQTS, BrS, short QT syndrome (SQTS), and WPW. Adding a molecular autopsy and family investigation enhance disease discovery in an additional 30–50 %.^34,35^ WPW remains a condition in which SCD and SCA occur as events that are rare but likely to occur more frequently in the young.^36^ Unless a SCD victim has had a previous ECG, which is uncommon in children, it is not possible to ascertain this as the etiology for a SCD. The detail needed to identify an accessory connection in an autopsy is rarely undertaken. In a multicenter study of SCA and SCD victims with WPW where there were ECG or electrophysiology study (EPS) data to confirm the diagnosis, a life-threatening event was the sentinel symptom in 65 %.^36^

Although efforts have been made by multiple organizations to screen children for cardiac conditions, the past emphasis has been on screening before athletic competition. In 2021 a comprehensive review of conditions that should prompt more attention and cardiology evaluation was published by the American Academy of Pediatrics emphasizing the role of the primary care provider in the evaluation of children. This identifies 4 main screening questions recommended, not just for athletes, but for all children, although to date there has not been consensus on testing that is required as part of screening. The modified 4 questions, based on expert opinion are: 1) Have you ever fainted, passed out, or had an unexplained seizure suddenly and without warning, especially during exercise or in response to sudden loud noises, such as doorbells, alarm clocks, and ringing telephones? 2) Have you ever had exercise-related chest pain or shortness of breath? 3) Has anyone in your immediate family (parents, grandparents, siblings) or other, more distant relatives (aunts, uncles, cousins) died of heart problems or had an unexpected sudden death before age 50? This would include unexpected drownings, unexplained auto crashes in which the relative was driving, or sudden infant death syndrome (SIDS). 4) Are you related to anyone with HCM or hypertrophic obstructive cardiomyopathy, Marfan syndrome, ACM, LQTS, SQTS, BrS, or CPVT or anyone younger than 50 years with a pacemaker or implantable defibrillator?^37^

### Diet-related factors

Certain dietary factors have been associated with risk for SCD. In some observational studies, consuming fish 1–2 times weekly was associated with 42 %–50 % reduction in SCD risk.^38–40^ This risk reduction may be partially related to intake of n-3 fatty acids (eicosapentaenoic acid and docosahexaenoic acid) found in fish.^41,42^ In addition, the risk for SCD has been reported to be lower with increasing dietary magnesium intake and increasing plasma magnesium levels. Each 0.25 mg/dL (1 standard deviation) increment in plasma magnesium concentration was associated with a 41 % reduced risk of SCD.^43,44^ Moreover, dietary patterns, such as the Mediterranean-style diet, have also been related to lower SCD risk among women.^45,46^ However, these findings have not translated to guideline-level recommendations regarding diet as a means of reducing SCA risk.

Alcohol consumption may have a complex effect on SCD risk. Prospective studies of individuals consuming ½ to 1 drink per day have shown reduced risk for SCD compared with nondrinkers.^47–49^ Consuming >1 drink/day was associated with twice the risk of experiencing VF during acute ST-segment-elevation myocardial infarction (MI) compared with ≤1 drink/day.^50^ Consuming >6 drinks/d was associated with increased risk for SCD.^51^ This increased risk of SCD could be related to increased risk of alcohol-induced cardiomyopathy that is seen in heavy drinkers and those with alcohol use disorder.^52^ In summary, the data suggest that high levels of alcohol consumption may have proarrhythmic effects and consequently increase SCD risk.

### SCD risk in those with a family history of SCD

Case-control studies have demonstrated that a history of SCD in a first-degree relative is an independent risk factor for VF and SCD, independent of CAD.^50,53,54^ The Paris Prospective Study showed that parental history of SCD was an independent risk factor for the occurrence of SCD (relative risk, 1.80; 95 % confidence interval (CI), 1.11–2.88).^31^ Genetics may predispose to SCD as a discrete trait, and/or may affect the manifestation of CAD. Screening for such genetic variants in patients with a family history of SCD and no established CVD may help to identify risk for SCD that may indicate closer surveillance and risk factor modification efforts.

### SCD risk in patients with structural heart disease

In patients with identified CVD, screening strategies have focused on identifying those who might benefit most from an ICD for primary prevention of SCD. In general, patients selected for primary-prevention ICD implantation should have a reasonable expectation of meaningful survival of at least 1 year.

### Ischemic cardiomyopathy

Ischemic cardiomyopathy (ICM) is defined as left ventricular dysfunction associated with at least 75 % narrowing of a major coronary artery or a documented history of MI.^55^ After MI, mortality risk increases gradually as LVEF declines to 40 %, and then exponentially increases as LVEF decreases further.^56^ The risk of SCA and SCD is especially high in this population. Studies have shown that the risk for SCD is greatest in the 30-day period post-MI and continues to remain high for many months after. In the Sudden Death in Patients with Myocardial Infarction study, Solomon et al. reported that the incidence of SCD or SCA with resuscitation was 1.4 % (95 % CI 1.2–1.6) in the first month after MI with a decrease to 0.5 % in months 1–6, 0.27 % per month in months 6–12, and 0.18 % per month in the 1–2 year period. The cumulative incidence of SCD or SCA with resuscitation was 7.5 % in the 2 year period after MI.^57^ Each decrease of 5 % in LVEF was associated with a 21 % adjusted increase in the risk of SCA/SCD in the first 30 days.^57^

#### ICD benefit in chronic ICM.

Per the 2022 AHA/ACC/HFSA guideline for the management of HF and the 2017 AHA/ACC/HRS guideline for management of ventricular arrhythmias (VA) and prevention of SCD, ICD therapy for primary prevention of SCD is recommended (Class I A) in patients with ICM who are at least 40 days post-MI and at least 90 days after coronary revascularization with LVEF ≤35 % and NYHA class II or III symptoms on chronic guideline directed medical therapy (GDMT).^58,59^ The results from the Multicenter Automated Defibrillator Implantation Trial (MADIT)^60^ and The Multicenter UnSustained Tachycardia Trial (MUSTT)^61^ that led to these recommendations are summarized in Table 3. ICD therapy is also recommended for patients with ischemic heart disease at least 40 days post-MI and 90 days post coronary revascularization with LVEF ≤30 % and NYHA class I symptoms on chronic GDMT (Class I B-R).^58,59^ The results from the Multicenter Automated Defibrillator Implantation Trial-II (MADIT-II)^62^ and the Sudden Cardiac Death in Heart Failure Trial (SCD-HEFT)^55^ that led to this recommendation are seen in Table 3.

The delay of 40 days post MI for primary prevention ICDs is based on the lack of an ICD mortality benefit in the Defibrillator in Acute Myocardial Infarction Trial (DINAMIT) and Immediate Risk Stratification Improves Survival (IRIS) trials.^63,64^ The delay of ICD implantation for 90 days after coronary revascularization comes from HRS/ACC/AHA consensus guidelines.^65^ The consensus guidelines state that revascularization has important time-dependent benefits and also highlights the exclusion and/or underrepresentation of patients within 90 days of revascularization in MADIT-I^60^, MADIT-II^62^ and SCD-HEFT^55^ trials. Furthermore, substudies of MADIT-II and SCD-HEFT show that an ICD has increasing survival benefit as time from revascularization increases.^66,67^

The current AHA/ACC/HFSA/HRS guidelines for primary and secondary prevention of SCD in patients with ischemic heart disease and major clinical trials are seen in Table 3.

### Non-ischemic cardiomyopathy

Non-ischemic cardiomyopathy (NICM) is defined as LV systolic dysfunction without marked coronary stenosis.^55^ Causes of NICM include genetic cardiomyopathies, myocarditis, autoimmune disorders, toxicity due to alcohol, chemotherapeutic agents, heavy metals, or other toxins, valvular disease, or electrical dyssynchrony. Two primary prevention randomized trials of ICD therapy included NICM patients with LVEF ≤35 % and HF symptoms (NYHA I–III), and demonstrated significant reductions in the SCD rate in patients with NICM randomized to ICD therapy. The results from the Defibrillators in Non-Ischemic Cardiomyopathy Treatment Evaluation (DEFINITE) trial^68^ and SCD-HEFT^55^ are summarized in Table 4. Based on these results, the AHA/ACC/HRS guidelines for management of patients with VAs and prevention of SCD give a class I recommendation for primary prevention ICD implantation in patients with NICM, HF (NYHA class II-III symptoms) and LVEF ≤35 % despite GDMT for at least 90 days.^58,59^

In a more recent study, Defibrillator Implantation in Patients with Nonischemic Systolic Heart Failure (DANISH), ICD therapy reduced SCD risk, but did not decrease the primary endpoint of total mortality.^69^ This finding has led to some calls for a reconsideration of primary prevention ICDs in patients with NICM. However, there are some aspects of the trial that could have mitigated the mortality benefit due to ICDs. The trial required an elevated N-terminal pro-b-type natriuretic peptide (pro-BNP) level for enrollment, which could have biased the results towards a higher risk of HF death than SCD.^69^ Additionally, 58 % of patients in each limb received cardiac resynchronization therapy (CRT).^69^ There was excellent use and adherence to GDMT in the trial which is often not the case in clinical practice, possibly further mitigating the mortality benefit of ICD in the study.^69^ Furthermore, a subgroup analysis of DANISH demonstrated mortality benefit with ICD therapy among patients younger than 68 years, suggesting that the absence of benefit of ICD in DANISH may be related to selection of an older population with higher rate of non SCD.^70^

### Inherited arrhythmogenic cardiomyopathies

Inherited arrhythmogenic cardiomyopathies is defined as an arrhythmogenic heart muscle disorder not explained by ischemic, hypertensive, or valvular heart disease.^73^ The etiology of ACM could be due to a systemic disorder (eg, sarcoidosis), an isolated cardiac abnormality (eg, myocarditis) or can be genetic.^73^ There are >40 genes that are known to be associated with inherited ACM. Some of these include *PKP2*, *LMNA*, *TTN*, *MYH7* and *TNNT2*.^74^ These genes encode various proteins involved in muscle structure, desmosomal function, ion channel function etc., and variants can cause both cardiac and extracardiac manifestations.^73^ An ACM patient typically presents with arrhythmia but the phenotype can overlap with other cardiomyopathies such as DCM where ventricular dilatation and systolic dysfunction may accompany the arrhythmia.^73^ Additionally, even within the broad category of ACM, there is often significant phenotypic overlap. The reason for this overlap is the fact that the mechanisms responsible for the phenotype rely on the dysfunction of final common protein pathways.^73^ Some of the most well recognized ACMs defined by their classic phenotype are discussed in this section.

#### Arrhythmogenic right ventricular cardiomyopathy.

Arrhythmogenic right ventricular cardiomyopathy (ARVC) is the best characterized of the ACMs and is most often familial, with autosomal dominant inheritance.^73^ There are various mutations in a number of genes encoding components of the desmosome that can cause ARVC, with mutations in Plakophilin-2 (*PKP2*) being the most common.^75,76^ ARVC is characterized by fibrofatty replacement of the right ventricular myocardium, though left ventricular involvement can also occur.^77^ SCA is common in patients with ARVC and it may be the first arrhythmic event in up to 50 % of cases.^78,79^ Current AHA/ACC/HRS guidelines give a class I recommendation for ICD for primary prevention of SCA in those with ARVC who have poorly tolerated sustained VT.^73^ The guidelines state that an ICD may be reasonable for those with ARVC and syncope suspected to be due to VA, and for those with hemodynamically tolerated sustained VT.^73^ Additionally, the guidelines also take into account the presence of certain VA risk factors including major (NSVT, inducibility of VT at EP study, LVEF ≤49 %) and minor (e.g. male sex, >1000 PVCs/24 h, RV dysfunction) ones. Per the 2019 HRS Expert Consensus Statement on ACM, an ICD may be reasonable for individuals with ARVC and three major, two major and two minor, or one major and four minor risk factors for VA.^73^ There are ongoing clinical trials, including RIDGE-1 (NCT06228924)^80^ and HEROIC-PKP2 (NCT06109181)^81^, testing gene therapies targeting ARVC patients with *PKP2* gene mutations. In preclinical studies, a single dose of the gene therapy restored healthy levels of PKP2 protein, normalized heart rhythms, improved right and left ventricular size and function and extended survival.^80^ The current ARVC guidelines are highlighted in Table 5.

#### Arrhythmogenic left ventricular cardiomyopathy.

Evaluation of patients with ARVC through postmortem investigations, genotype-phenotype correlation studies and cardiac MRI (CMR) led to awareness that the disease often also involves the LV.^82–84^ This led to the identification of individuals and families with early and/or predominant LV arrhythmia and structural abnormalities, leading to increasing recognition of arrhythmogenic left ventricular cardiomyopathy (ALVC) as a separate disease entity.^73^ Mutations in a few desmosomal genes such as Desmoplakin (*DSP*), Phospholamban (*PLN*) and Filamin C (*FLNC*) have been associated with ACM with predominantly LV systolic dysfunction.^82^ Studies have shown that *DSP* cardiomyopathy involved the LV in almost all cases, and often without any RV involvement.^85^ The primary clinical signs of *DSP* cardiomyopathy are LV systolic dysfunction, LV fibrosis on MRI, and frequent PVCs. In comparison to classical ARVC phenotypes, higher, similar, or lower VA rates have all been reported for patients with *DSP* variants.^86^ A specific management strategy for SCD prevention in ALVC is yet to be formulated.^82^ ALVC patients currently fall within the general ICD guidelines for patients with ACM (other than ARVC). These are highlighted in Table 5.

#### Lamin A/C cardiomyopathy.

Lamins are proteins that line the inside of the nuclear membrane of cells and confer mechanical stability.^87^ Lamin A/C (*LMNA*) mutation mediated cardiomyopathies (Laminopathies) are a well-recognized group of inherited ACMs that are characterized by atrial fibrillation and cardiac conduction disease, which can precede the development of VAs and cardiomyopathy by decades.^73,88^ They are usually inherited in an autosomal dominant fashion and cause familial DCM^89^, either in isolation, or in association with skeletal muscle dystrophies such as Emery-Dreifuss muscular dystrophy^90^ and autosomal dominant limb girdle muscular dystrophy.^91^ However, *LMNA* variants have also been identified in patients diagnosed with ARVC, again highlighting the genetic overlap in inherited ACMs.^92^ The optimal age for initiating screening of family members of *LMNA* cardiomyopathy patients and risk stratification for SCD is still not well defined.^93^ SCD occurs frequently in laminopathies and often precedes development of cardiomyopathy,^94^ with some studies reporting as high as 46 % of deaths being due to SCD.^95^ Societal guidelines give a class IIa recommendation for ICD implantation in patients with cardiomyopathy due to *LMNA* mutation with 2 or more risk factors; history of nonsustained ventricular tachycardia (NSVT), LVEF <45 %, non-missense mutation and male sex.^58,59,96,97^ See Table 5.

### Hypertrophic cardiomyopathy

HCM, defined by increased left ventricle (LV) wall thickness not solely explained by abnormal loading conditions, is the most common inherited cardiac disease.^98^ To date, >1500 mutations in >11 genes that code for sarcomere proteins have been identified.^98–100^ SCA is common in HCM patients and represents a large proportion of their 0.5–2 % annual incidence of cardiovascular death. The 2024 ACC/AHA/HRS guideline on the management of patients with HCM gives a class I recommendation for ICD placement for patients with HCM and previous documented cardiac arrest or sustained VT.^99,101,102^ Additionally, they give a class IIa recommendation for offering an ICD to adult patients with HCM with ≥1 major risk factor: sudden death definitively or likely attributable to HCM in ≥1 first-degree or close relatives who are ≤50 years of age, massive LVH (≥30 mm in any LV segment), syncope suspected by clinical history to be arrhythmic, LV apical aneurysm with transmural scar or late gadolinium enhancement (LGE) on CMR, and LVEF <50 %.^99^ Lastly, they give a class IIb recommendation for consideration of ICD in select adult patients with HCM and without major SCD risk factors but with extensive LGE by CMR or NSVT present on ambulatory monitoring.^99^ The current guideline recommendations for the primary and secondary prevention of SCD in HCM patients are highlighted in Table 5.

### SCD risk in patients with inherited channelopathies

Inherited arrhythmia disorders include conditions that are associated with inherited channelopathies or inherited structural heart disease. In contrast to previously mentioned inherited conditions such as ACMs and HCM that increase SCD risk with structural abnormalities, patients with inherited channelopathies have electrically abnormal, but structurally normal hearts. The most well-known inherited arrhythmia disorders include LQTS, BrS, CPVT and SQTS. These conditions have varying annual incidences of cardiac events (unexplained syncope, aborted cardiac arrest requiring cardiac resuscitation, unexpected sudden death) ranging from 0.3 to 0.6 %/year in some forms of LQTS^103^ to 8-year rates of SCA/SCD of up to 13 % in CPVT.^104^ Due to the lack of randomized and/or blinded studies, most available data on inherited arrhythmic disorders are derived from patient registries.^105^ In general, current AHA/ACC/HRS guidelines recommend secondary prevention ICD therapy for patients with known channelopathies and a history of SCA.^58^ However, in CPVT a high risk of ICD related complications and the effective medical therapies make this recommendation less clear in this population.^106^

### Long QT syndrome

LQTS is the most common inherited channelopathy estimated to occur in 1 in 2500 persons. In most cases, its inheritance is autosomal dominant.^107^ Three major causative genes for various forms of LQTS include, *KCNQ1, KCNH2*, and *SCN5A*. *KCNQ1* and *KCNH2* encode subunits of potassium channels and *SCN5A* encodes a subunit of a cardiac sodium channel.^108^ These variants affect membrane potential and, therefore, the cardiac action potential. LQTS is typically characterized by a prolongation of the QT interval on the ECG and by the occurrence of syncope or SCA. The provocation can be emotional or physical stress in LQT1, auditory stimulation and postpartum events in LQT2 and sleep and bradycardia in LQT3.^107^ T-wave alternans on ECG can identify patients at a particularly high risk for SCA. Additionally, sinus pauses unrelated to sinus arrhythmia are also a warning sign.^107^ The ventricular tachyarrhythmia that underlies the cardiac events of LQTS is torsades de pointes, which can be self-limiting and produces transient syncope but can also degenerate into VF and cause SCA.^107^ β-Blockers are the first-line therapy for LQTS.^107^ This recommendation comes from a 1985 study which included 233 symptomatic LQTS patients and showed a significant positive impact on survival from pharmacologic or surgical antiadrenergic therapy.^109^ Nonselective β blockers (nadolol and propranolol) are recommended and there are data to support the use of mexiletine in LQT3 in whom β-Blockers may not be effective^110^, and in highly symptomatic LQT2.^111^ For patients with LQTS, the current AHA/ACC/HRS guidelines give a class I recommendation for primary prophylactic implantation of an ICD in high risk patients who are unresponsive or intolerant to β blockers. High-risk patients include those with QTc >500 ms, long QT type 2 and 3, females with long QT type 2, age < 40 years, onset of symptoms age < 10 years and patients with prior cardiac arrest or recurrent syncope. For asymptomatic LQTS and a resting QTc >500 ms (and often cited 550 ms in LQT1) while on optimal beta blocker therapy, there is a class IIb recommendation for ICD placement.^58^

### Brugada syndrome

BrS is an autosomal dominantly inherited arrhythmia disorder in cases where a pathogenic *SCN5A* variant is identified but this is only about 25 % of the BrS population. The remainder of patients likely have polygenic or oligogenic disease. BrS has a higher prevalence in males (8 to 10 times more in males after puberty)^112^ and a higher prevalence in parts of East Asia, due to specific prevalent genetic variants.^113^ The most common implicated gene is *SCN5A*, in which >100 mutations causative for BrS have been discovered.^105^ The pathophysiologic mechanism is either a decrease in the inward sodium or calcium current or an increase in one of the outward potassium currents.^105^ Presenting symptoms can include palpitations, chest discomfort, syncope and VF or aborted SCD. Symptoms usually occur at rest or sleep but rarely during exercise and manifest in adulthood with a mean age of SCD of 41 ± 15 years.^112^ BrS is definitively diagnosed when a type 1 Brugada pattern, with a downward sloping, coved ST-segment elevation of 2 mm or greater, is observed either spontaneously or after intravenous administration of a sodium channel blocking agent (eg, procainamide) in at least one right precordial lead (V1 and V2). For patients with BrS, guidelines give a class I recommendation for ICD placement in patients with a type I ECG and history of cardiac arrest, sustained VA or syncope likely caused by VA.^58^

### Catecholaminergic polymorphic ventricular tachycardia

CPVT is a rare arrhythmogenic disorder characterized by adrenergic-induced bidirectional and polymorphic VT.^105^ CPVT1 and CPVT2 are the two most common types of CPVT that have been identified, which result from mutations in the cardiac ryanodine receptor (*RyR2*)^114^ and cardiac calsequestrin (*CASQ2*)^115^ genes, respectively. The first clinical manifestation is usually syncope which can manifest in the first or second decade of life and which is usually prompted by exercise or emotional stress. A family history of syncope may also be present in 30 % of cases.^105^ The resting ECG is usually normal, occasionally with bradycardia.^104^ Once patients start exercising, ventricular ectopy develops, increasing in complexity as the heart rate increases. Atrial arrhythmias are also seen. Therefore, Holter monitoring, implantable loop recorders (ILRs) and exercise stress tests help establish the diagnosis.^105^ Patients with CPVT are at high risk for SCD without treatment.^104^ The first-line therapy is β -blockers without intrinsic sympathomimetic activity combined with exercise restriction.^105^ The high burden of inappropriate ICD shocks and ICD storm in CPVT has led to a more guarded approach and advocates for restraint from ICDs with use of triple therapy involving β blockade with nadolol, flecainide, and left cardiac sympathectomy in the highly symptomatic population.^116^ If the above measures fail, the guidelines give a class I recommendation for treatment intensification with an ICD if there is recurrent sustained VT or syncope.^58^ Gene therapies targeting *CASQ2* have been evaluated in rodent models and have shown that transfer of wild-type *CASQ2* prevents and reverts severe manifestations of CPVT and that this curative effect lasts for 1 year after a single injection of the vector.^117^ However, gene therapies for CPVT have not been studied in human trials yet but studies are about to commence.

### Short QT syndrome

SQTS is one of the rarer cardiac channelopathies and a hallmark sign of this disease is a short QT interval.^105^ DNA variants in 3 potassium channel genes (*KCNH2, KCNQ1, KCNJ2*) have been associated with SQTS.^118–120^ Mutations in the same genes cause variants of LQTS as well. However, while LQTS is typically caused by loss-of-function mutations, SQTS is caused by gain-of-function mutations.^105^SQTS has also been associated with loss-of-function mutations in L-type cardiac calcium channel genes (*CACNA1C* and *CACNB2*).^121^ Expert consensus guidelines state that a cutoff value of QTc ≤330 ms should be used for the diagnosis of SQTS. The guidelines give a class I recommendation for ICD implantation in those who have cardiac arrest or sustained VA.^58^ The optimal strategy for primary prevention of SCA in SQTS is unclear given the lack of identifiable independent risk factors for SCA.^105^

The current guidelines for the primary and secondary prevention of SCD in patients with primary inherited arrhythmia syndromes are included in Table 5.

## SCD prediction challenge: moving beyond LVEF

Severe left ventricular dysfunction is currently the most common indication for implantation of an ICD for the primary prevention of SCD.^60,62^ However, LVEF is an imperfect tool for SCD risk stratification due to limitations in three main areas: scope, sensitivity, and specificity.

**Fig. 2.**
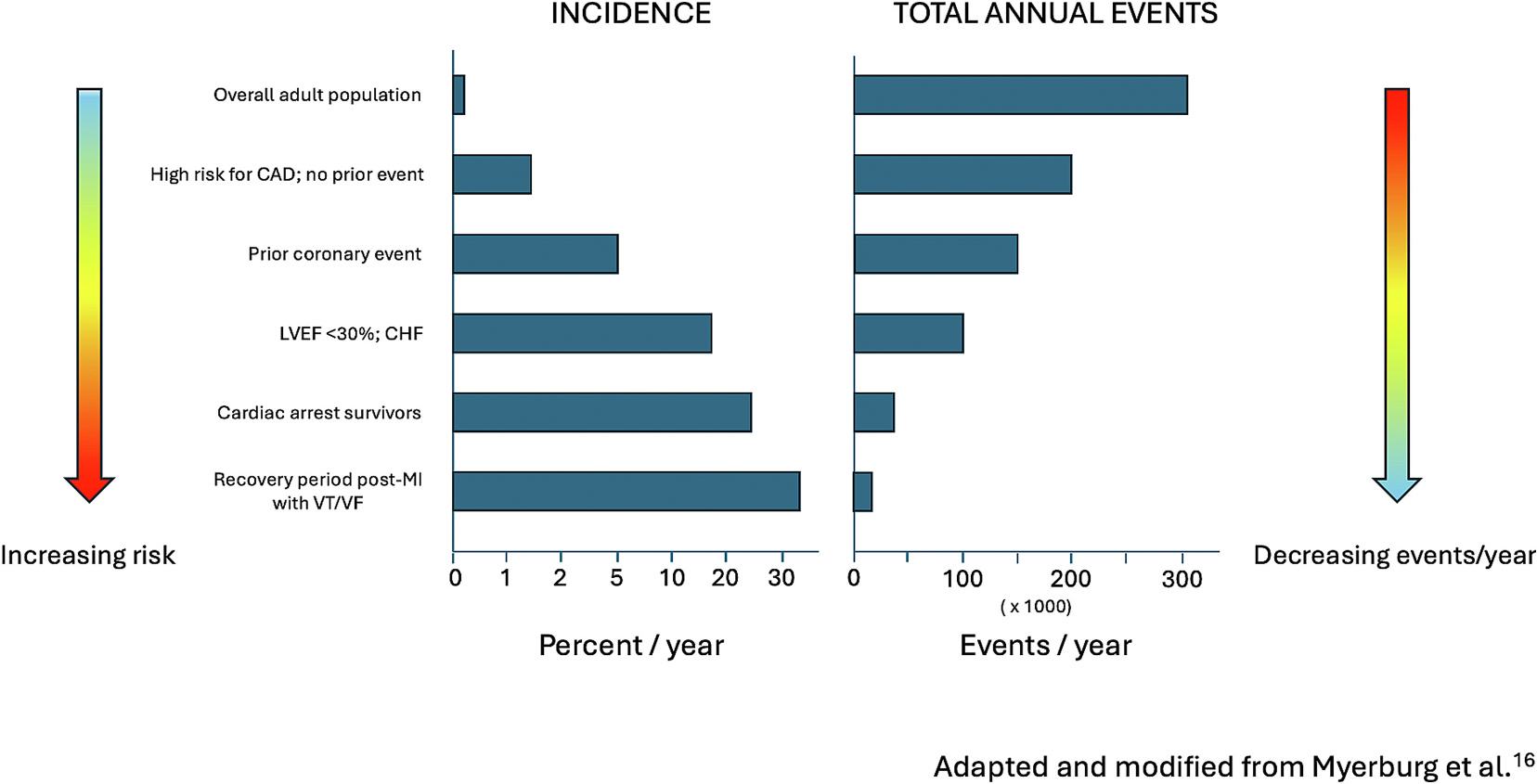
Comparison of SCD Annual Risk vs. Total Annual Events by Clinical Subgroups.

In terms of scope, although the subgroup of patients with low LVEF have a high SCD incidence per year, only a small proportion of the total number of SCD events per year occur in this subgroup.^19^ As first highlighted by Myerburg et al., the absolute number of individuals at risk (and consequently the number of annual SCDs) decreases as one moves from low risk to high risk subgroups, with the greatest number occurring in the general population who are at lowest individual risk.^19^ (Fig. 2). Although the annual incidences of SCD in subgroups of the population have changed over the years (for example, the incidence of SCD post-MI has declined with time, with rates less than 1 % per year in patients on optimal medical therapy and revascularization^13,122,123^), the overarching challenge remains the same. Solely focusing SCD preventive efforts on patients with a low LVEF is not a great strategy from a public health perspective, as it does not take into account the majority of SCD patients who fall into lower risk groups and do not meet criteria for ICD placement.

Another important shortcoming of low LVEF as a SCD risk stratification tool is low sensitivity. Retrospective studies of SCD cases have shown that among patients who had their LVEF assessed prior to death, only 20–30 % qualified as candidates for a prophylactic ICD,^124,125^ suggesting that 70 % of SCDs occurred in patients who fall outside the current recommendations for prevention.^126^ Using the MUSTT trial data, risk-stratification algorithms constructed to weight the prognostic impact of various clinical variables on the risks of arrhythmic death and total mortality showed that the variables with the greatest prognostic impact included LVEF, age, NYHA functional class, history of heart failure, NSVT, left ventricular conduction abnormalities, inducible sustained VT, and atrial fibrillation.^127^ Among these, inducible VT, history of heart failure, LVEF, and left bundle branch block (LBBB) or intraventricular conduction delay (IVCD) had statistically significant associations with the end point of SCA/SCD. Surprisingly, their multivariable model showed that patients whose only risk factor was LVEF ≤30 % had a predicted 2-year risk of SCD of less than 5 %.^127^ The risk of SCD increased as additional risk factors were added. Importantly, these authors also found that patients with LVEF >30 % and other risk factors may have higher mortality and a higher risk of SCD than some patients with LVEF ≤30 % and no other risk factors.^127^ Overall, their findings suggest that the use of LVEF ≤30 % as a sole criterion for patient selection for primary prevention ICDs may not be ideal.

Low LVEF also faces the issue of low specificity due to competing risks, as it does not have the ability to specifically predict risk for SCD versus non-sudden death (non-SCD).^128^ This is an important distinction, since patients destined for non-SCD would not benefit from ICD placement. Using data from MADIT II,^62^ Goldenberg et al. developed a clinical risk score for the end point of all-cause mortality.^129^ The score was derived from 5 variables (age, NYHA functional class, blood urea (BUN), atrial fibrillation, and QRS duration) which they identified as being highly predictive for all-cause mortality, each given a score of 1. Among the conventional therapy group, mortality rates were noted to increase with increasing risk score and the mortality benefit in the ICD risk group showed a U-shaped pattern, wherein there was no apparent mortality benefit of the ICD among patients with 2-year mortality rates that were very low (risk score 0) or very high (risk score 3+). Of note, in the conventional therapy group, the rate of SCD increased with increasing risk score from 0 to 2 (around 5 %, 10 % and 15 % for groups 0,1 and 2 respectively). The increase in SCD risk was attenuated for the higher risk groups of 3+, while non-SCD was noted to be high. Accordingly, the benefit of ICD was greatest in the intermediate risk groups, who had substantial increases in the rates of SCA. The rates of appropriate ICD therapy were also noted to vary across the groups. In the 0-risk group,18 % experienced appropriate ICD therapy during an average follow-up time of 21 months, as compared with 26 % of the patients with ≥1 risk factor (*p* = 0.04).^129^ In the more contemporary DANISH study, rates of ICD therapy were also low. Out of 556 patients randomized to receive an ICD, termination of VT by antitachycardia pacing (ATP) occurred in 17.4 % in the ICD group, and appropriate shock for VF or VT occurred in 11.5 % in the ICD group.^69^ While the authors noted that their study population consisted of older patients with higher risk for non-SCD, they still emphasized the need for developing risk scores to identify NICM patients truly at higher risk for arrhythmia to avoid implantation of ICD in lower-risk patients who may not benefit from it.^69^ Overall, these studies highlight that even among patients who meet the criteria to receive an ICD based on LVEF ≤30 % alone, there is significant heterogeneity in the rates of SCD, non-SCD, and appropriate ICD therapy, which could be accounted for by the incorporation of other predictive variables.

### Novel approaches to SCD prediction

In summary, the above studies suggest that low LVEF as a sole selection criteria for ICD placement fails to capture a considerable number of SCD patients who do not meet the cut-off, while at the same time, the majority of patients who receive an ICD, may never receive an appropriate shock. Therefore, the medical community is challenged with the task of addressing this problem by formulating more sensitive and specific models for prediction.^11^

There have been other efforts to develop more specific models for SCD prediction. The Seattle Proportional Risk Model calculates SCA risk by taking into account other clinical and laboratory variables in addition to LVEF.^130,131^ More recently, a 13-variable ventricular fibrillation risk score (VFRisk) was developed and validated in the Oregon and California community-based studies, that improved the C-statistic for SCA risk from 0.638 (95 % CI: 0.598–0.678) based on LVEF ≤35 % alone to 0.808 (95 % CI: 0.774–0.842).^132^ Scar by cardiac MRI (cMRI) or other advanced imaging techniques may be an important risk marker for SCD and could potentially help to risk stratify patients better than LVEF alone. For example, the absence of scar in NICM identifies patients at low risk for SCD^133,134^, and increased scar burden in ischemic cardiomyopathy identifies patients at higher risk for SCD.^135^ Scar assessment by MRI has been incorporated into various guidelines for SCD risk assessment.^136^ However, access to MRI may be variable and disparate, and is not necessarily appropriate as a screening test for overall low-risk populations.

Thus, although additional data from clinical variables and imaging such as cMRI may pave the way forward to developing more tailored approaches for SCD risk stratification, particularly in patients with identified structural heart disease, these approaches must address the availability of such advanced and costly imaging modalities and disparities in access to such technologies.

## Improving SCD prediction: the role of artificial intelligence

As approaches beyond LVEF are necessary to improve SCD prediction in those with known CVD, and as the majority of SCD cases occur in people with no known cardiac disease,^13^ the following sections will explore avenues to improve SCD prediction in both of these groups and examine the role that AI could play in helping us to advance SCD prediction.

### Warning symptoms and the role of near-term prediction of SCA

In the context of SCA, the phrase near-term prediction is typically used to refer to a period of minutes to days leading up to the event. In approximately half of patients who suffer SCA/SCD, there are warning symptoms, such as angina, that precede the event.^11,137^ In the Oregon Sudden Unexpected Death Study (Oregon-SUDS), a community-based prospective study of out-of-hospital SCA, a comprehensive assessment of warning symptoms in the four weeks prior to SCA revealed that 51 % of patients presented with at least one symptom in the 4 weeks leading up to the event. Most patients had a recurrence of symptoms during the 24 h prior to SCA. Of these, 80 % had symptom onset more than one hour prior to the event. The main symptoms were chest pain (46 %), abdominal symptoms (20 %), dyspnea (18 %), and syncope (5 %), with significant overlap, especially between chest pain and dyspnea. Only 19 % of patients with symptoms called 911. Among patients with symptoms who did summon emergency medical services (EMS), 78 % developed SCA before EMS arrival, whereas 22 % experienced SCA in the ambulance. Patients who called 911 were more likely to have had a witnessed arrest, had a higher proportion of bystander CPR, and more often presented with a shockable rhythm. Survival to hospital discharge was 32.1 % for those who called 911, compared with only 6.0 % for those who did not call 911.^138,139^

This study suggests the potential utility of SCA risk stratification strategies based on reported symptoms in the days to weeks leading up to the event. In a follow-up study designed to include a comparison group of non-SCA 911 callers, Reinier et al. reported that symptoms preceding imminent SCA were sex-specific and additional features were needed to improve prediction using warning symptoms.^140^ Further investigation is necessary to determine exact symptom-based risk stratification algorithms for these patients and to determine the causal associations between early 911 calls and improved survival. Strategies to minimize false-positive alarms will also be required to optimize resource utilization.

### Artificial intelligence-based prediction of SCA and fatal ventricular arrhythmias

AI typically refers to the overarching field which involves using techniques like machine learning (ML) and deep learning (DL) to create intelligent machines. ML is a specific approach within AI that uses algorithms that allow machines to learn from data without explicit programming, helping them identify patterns. DL is a subset of ML. While traditional ML algorithms need data with explicit feature engineering, DL models can automatically learn features from raw unstructured data such as images, audio etc.^141^ For the purposes of the following sections, AI will refer to the all-encompassing field including its various techniques. Specific techniques such as ML and DL are mentioned if the studies mentioned specifically utilized them.

The pathophysiology of SCA can be complex and involve non-linear relationships between multiple contributing risk factors (CAD, diabetes, HTN, inherited conditions, etc.) and acute triggers (ischemia, electrolyte imbalances, etc). Given such a multifactorial pathophysiology, conventional risk stratification methods often prove inadequate or laborious to use manually. On the other hand, ML algorithms can use large datasets to identify inapparent predictive patterns.^142^ Several AI-based algorithms for SCA prediction have been developed using an array of input data, from cMRI to clinical history. Many of these algorithms have outperformed traditional prediction models such as LVEF, albeit with their own limitations. AI seems likely to play a crucial role in advancing the field of SCA prediction.

### AI in long-term prediction

Some AI algorithms developed using diverse input data have shown promise in the long-term prediction of SCA (i.e., up to months to years prior to the event). As a point of reference, previous studies in various populations have estimated the receiver-operator characteristic (ROC) curve’s area under the curve (AUC) for LVEF in the long-term prediction of SCD to be 0.59–0.68.^143,144^ Many of the AI models discussed below report a higher AUC than LVEF. However, most of these models lack external validation, and focus only on patients with severe LV dysfunction and/or an existing ICD, which limits the models’ generalizability.

#### ECG-based models.

ECG-based AI deep learning models (AI-ECG) have shown promise in the detection of diseases such as atrial fibrillation^145^, cardiac amyloidosis^146^, screening for LV dysfunction^147^, and prediction of 1-year all-cause mortality.^148^ In patients with heart failure, Shiraishi et al., showed that the combination of AI-ECG and conventional SCD risk predictors (LVEF and NYHA class) achieved a higher AUC than LVEF alone (0.66 vs 0.59, *p* = 0.017).^143^ Holmstrom et al. developed an AI-ECG model for prediction of SCD risk in the Oregon-SUDS which was successfully replicated in their southern California study. This DL model achieved an AUC of 0.889 (95 % CI 0.861–0.917) for the detection of SCD cases vs. controls, performing significantly better than a conventional ECG-marker model that achieved an AUC of 0.712 (0.668–0.756).^149^ Pham et al. examined whether dynamic ECG changes are associated with an increased risk for SCD. The risk score looked at the following 6 features: heart rate > 75 bpm, QRS transition past V4, LVH, frontal QRS-T angle >90, QTc interval > 450/460 ms and T peak-T end interval > 89 ms. They found that SCD patients displayed a greater electrical risk score increase compared with controls, with their multivariable model incorporating the change in ECG risk score demonstrating a higher AUC compared with a model that only included baseline ECG, demographics and clinical SCD risk factors (0.87 vs.0.77).^150^ Incorporating such dynamic ECG changes in AI models could yield superior SCD prediction results.

#### Cardiac MRI-based models.

Scar tissue formation and remodeling of the heart result in regional heterogeneities in the electrophysiological properties of the myocardium,^151^ creating the substrate for the initiation and maintenance of VAs in diseased hearts.

Arevalo, et al., used cMRI images in a proof-of-concept study to create personalized three-dimensional computer models of post-infarction hearts and tested each model’s propensity for ventricular arrhythmia. Pixels within the 3D model were classified as scar tissue where appropriate and they were assigned fiber orientations and electrophysiological properties. Importantly, myocytes in the infarct border zone were assigned remodeled action potential dynamics (extended duration compared to non-infarcted tissue). Subsequently, the virtual models were paced from a number of locations within the ventricle and patients were classified as being at risk for SCD if arrhythmia was elicited from at least one of the pacing locations. In their cohort, their model was superior to LVEF in predicting SCD.^152^ However, the processing and analysis of the cMRI in their computational model are time intensive, rendering it impractical in the screening of large populations.

DL models, on the other hand, do not require extensive processing of cMRI images. They are often able within seconds to analyze raw images and combine them with other clinical covariates to estimate SCD probability. Popescu et al. developed one such DL model that used cMRI and other clinical covariates of patients with ischemic cardiomyopathy to estimate the probability of SCD within 10 years. The model was both internally and externally validated and achieved an AUC of 0.87 and 0.72 when applied to internal and external datasets, respectively. In addition to predicting the probability of SCD, the model was able to develop patient-specific survival curves and to predict time to SCD.^153^ Similarly, Okada et al., developed a ML model that used a profile of spatial complexity of myocardial tissue (generated from cMRI grayscale images) to predict appropriate ICD shock or SCD in patients with ischemic cardiomyopathy and LVEF ≤35 % who underwent ICD placement. The model achieved an AUC of 0.72, though the model was not externally validated and also required significant processing of the cMRI images, which can be resource limiting in most clinical settings.^154^ Another study used a DL model based on cine-cMRI images of patients who received primary prevention ICDs to derive a risk score for appropriate ICD therapy over median follow up of 7.1 years. The model achieved an AUC of 0.69.^155^

#### Other clinical models.

Nakajima et al. developed a DL model that used 123I-metaiodobenzylguanidine (l-MIBG) planar images of the heart to obtain the 123I-MIBG heart/mediastinal ratio (HMR) in patients with CHF (both reduced and preserved EF), and applied this along with 13 other variables, including age, gender, NYHA functional class and LVEF, to differentially predict the probability of life-threatening arrhythmic events vs. death due to progressive heart failure. The probability of heart failure death significantly increased as HMR decreased. However, the probability of arrhythmic events was maximal when HMR was intermediate (1.5–2.0 for patients with NYHA class III). The AUC of the model for predicting SCD and appropriate ICD shock or ATP was 0.8.^156^

Wu et al. used demographics combined with clinical characteristics (including laboratory values and cardiac image indices) of 382 ICD recipients (with LVEF ≤35 %) to develop an ML model to predict appropriate ICD therapy or suspected SCD during a follow-up time of 5.9 ± 2.3 years. Their model achieved an AUC of 0.88, the highest on record.^157^ However, the study had limited power to detect small differences in risk predictions due to the small number of primary events. Additionally, the model was not validated on an external dataset and the authors stated that external validation using a dataset with a larger number of primary events would be needed to truly test the predictive ability of the model.^157^

A summary of the above models and their AUCs is listed in Table 6.

### AI prediction in HCM and inherited arrhythmia disorders

Patients with conditions such as HCM, DCM, LQTS, BrS usually have different clinical characteristics and cardiac phenotypes (e.g., younger age, less likely to have CAD) compared with the vast majority of SCA patients (e.g., older and higher likelihood of CAD).^158^ Therefore, it is essential to develop focused risk stratification tools for these populations. In contrast to other broad AI-based prediction models for patients with SCD, the challenge for model generalizability comes from the complex phenotypes that result from acquired cardiac conditions, the small available sample sizes in HCM and inherited arrhythmia disorders and the variable disease expression in these genetic conditions.

ML models for predicting SCD and VAs are most advanced in the study of HCM. Smole et al., used multiple clinical variables, including physical examination, genetics, and imaging features, in around 2000 HCM patients, to train a ML model to predict the 5-year risk for the occurrence of various events such as VT, SCD, HF and appropriate ICD shock. Their model achieved an AUC of 0.70 for SCD and outperformed previously established models with an AUC of around 0.6.^159^ Alis et al. developed ML models for VT prediction based on cMRI images of HCM patients. Their best performing model achieved an AUC of 0.92.^160^ Another study used 22 clinical covariates (including clinical history, cardiac imaging, and medication) and achieved an AUC of 0.83 for prediction of sustained VT or VF in HCM patients.^161^ Lyon et al. used ML to identify high-risk ECG phenotypes in HCM patients, and found that primary T wave inversion with normal QRS was associated with the highest SCD risk score.^162^

These models were limited by few primary events and lacked external validation. External validation would be necessary to estimate the actual AUCs of these models. A summary of the above models is listed in Table 7.

### AI for near-term Prediction of out-of-hospital SCA

AI can potentially be used to predict SCA in the near-term in certain subsets of outpatients who have cardiac rhythm monitoring devices in place. Several studies have sought to develop ML models for predicting SCA within 2 min to 4 h before the event using 24-h ECG recordings from Holter monitors of SCD patients.^163,164^ Shi et al. studied 23 24 h-ECG recordings before SCD onset as well as a few seconds later of patients from the PhysioBank MIT/BIH Normal Sinus Rhythm (NSR) and MIT/BIH SCD databases.^165^ The patients from the SCD database had Holter monitors in place because they were deemed high risk for SCD due to their history of MI or tachyarrhythmia. Using ML algorithms on heart rate variability (HRV) data from the ECG signal 14 min prior to SCD (and a random 14-min interval for NSR patients), they decomposed the signal into time-domain, frequency-domain and entropy features. Comparing a combination of these features between NSR and SCD patients, they were able to predict SCD 14 min before onset and differentiate it from NSR with 96.1 % accuracy (95 % sensitivity and 97.2 % specificity).^165^ Panjaitan et al. also used HRV data from the same databases and other additional ones and used DL to predict SCD 30 min prior to onset.^166^ The model was tested using a validation dataset from the same database. The model achieved an accuracy of 99.3 % for predicting SCD, reaching 97 % sensitivity and 99.6 % specificity.^166^ Instead of HRV data, one study used the actual ECG signal data from the MIT/BIH NSR and SCD databases to create a model that was able to predict SCD 30 min before onset with an accuracy of 95.3 %.^164^

Apart from ambulatory ECG monitoring data, implanted devices such as pacemakers and ICDs are also important repositories of data. Ginder et al. conducted a post-hoc analysis using data from the IMPACT trial and studied 59,807 device transmissions from 2413 patients with ICDs and cardiac resynchronization therapy with defibrillators (CRT–Ds), in which appropriate ICD therapies were delivered to 151 patients (141 shocks and 10 ATPs).^167^ By using ML, they were able to identify patients at risk of VT or VF within 30 days of the event. They used 80 % of the dataset to train the model and 20 % of the dataset for validation. The ML model achieved an AUC of 0.98 and 0.90 for the training and validation datasets respectively.^167^

These studies suggest that the incorporation of AI algorithms into ambulatory ECG monitors and implanted devices such as pacemakers and ICDs could help healthcare providers identify patients at imminent risk for SCA through remote monitoring and develop strategies to mitigate SCD risk.

### AI for near-term prediction of in-hospital cardiac arrests

Approximately 292,000 in-hospital cardiac arrests (IHCA) occur in the United States each year, and the rate of survival to discharge for such patients is <20 %.^168,169^ In most cases of IHCA, patients have abnormal vital signs during the preceding 4 h.^170^ Rapid response systems are widely in use at various hospitals to triage patients for appropriate escalation of care depending on abnormalities noted in vital signs. Based on this, the modified early warning score (MEWS) has been developed, and has become one of the most widely used scores to predict clinical deterioration and cardiac arrest.^171^ However, scores such as MEWS have their limitations, such as low sensitivity and high false-alarm rates.^172^ Studies have shown that ML models based on similar clinical and laboratory parameters can outperform conventional models such as MEWS.^173–175^

Kwon et al. developed a DL-based early warning score (DEWS) that trained on the vital signs of around 52,000 patients (with 419 IHCAs) and achieved an AUC of 0.85, compared with the MEWS, which had an AUC of 0.63.^176^ This extended model also performed even better than MEWS on an external dataset (AUC 0.9 vs 0.785 respectively).^177^ The advantage of ML models over conventional models such as the MEWS lies in their ability to function on a wide range of input data, rather than solely relying on numerical vital signs data. Wu et al. developed a ML model trained on clinical features of 166 acute coronary syndrome (ACS) patients, such as clinical history, lab values, and vital signs collected 24 h before IHCA. The model achieved an AUC of 0.958 compared with the AUC of MEWS, which was 0.673. However, this model was not externally validated.^178^ Yet another study used demographics and ECG waveforms to train a ML model to predict IHCA within 24 h. During internal and external validation, the AUC of the model for predicting IHCA within 24h were 0.913 and 0.948, respectively. Additionally, the high-risk group identified by the model showed a significantly higher hazard for delayed cardiac arrest (5.74 % vs. 0.33 %, *P* < 0.001). Their results also indicated that IHCA could be predicted not only using a conventional 12-lead ECG but by using only a single-lead ECG.^179^ This suggests that ML models could potentially utilize the typically single-lead ECGs generated by wearable devices to predict IHCAs.

As powerful as MEWS was for predicting IHCAs, compared with out-of-hospital cardiac arrest (OHCA), IHCAs have a higher likelihood of non-cardiac etiologies.^180^ Conventional scores such as MEWS cannot discriminate between arrhythmic and other etiologies of IHCA. ML models, on the other hand, can draw from additional parameters to specifically predict arrhythmia. Wang et al. used demographics, medical history data, lab values, vital signs, and medications of around 2700 hospitalized patients with heart failure, to train a ML model to predict malignant arrhythmias (VT or VF). The model achieved an AUC of 0.867.^181^ Another study used ML to analyze HRV and respiratory rate variability (RRV) data collected from the monitors of patients admitted to the cardiac ICU to predict VT 1 h prior to the event. The study used recordings from 52 patients with VT and 52 controls and achieved an AUC of 0.93.^182^ The ability of such models to specifically predict arrhythmic vs non-arrhythmic arrest in hospitalized patients could be useful in guiding the healthcare team to implement targeted measures for such patients and prompt a speedier response if/when the arrhythmic event eventually occurs.

AI can also be utilized in the triage of emergency department (ED) patients to determine those who are at high risk for deterioration and therefore may benefit from intensive monitoring or early intervention. ML-based models utilizing simple parameters such as vital signs and HRV have outperformed MEWS in the prediction of IHCA in ED patients.^183–186^

Although the high AUCs of the above models seem promising, they should be interpreted cautiously. Almost all of the above models were limited by a small number of primary endpoints and were not separately validated using an external dataset. In order to truly state whether such prediction models could be relied upon to predict IHCA in day to day clinical settings, the models will need to be trained on larger datasets with higher numbers of cardiac arrest cases and then externally validated in large datasets as well. Furthermore, clinical testing would be necessary to determine whether the incorporation of such ML-based patient triage models can truly translate into improved outcomes in IHCA cases. A summary of the above models and their AUCs is listed in Table 8.

### Future directions for AI-based SCA prediction

Most existing ML models have been trained on and tested in specific subgroups of SCD patients with known CVD such as heart failure, ACM, and CAD. Therefore, these models do not address the largest subgroup of SCD cases, which occur in the prehospital period among patients without prior known CVD. The relatively simple vital signs-based ML models that have been used to predict IHCA cannot be extended to more long-term prediction of OHCA because of the different settings during which the models were developed. However, there are other ways in which AI could assist with the identification of those at risk for OHCA.

We do not have robust information regarding the proportion of patients who have had some form of established healthcare prior to their arrest. However, since the majority of SCD cases occur in older people, it is reasonable to assume that a significant number of those patients have seen a healthcare professional or had a routine workup at some point prior to their arrest. Consequently, increasing the diagnosis of cardiovascular conditions, perhaps through AI’s ability to detect subtle signs that are typically missed in the outpatient setting, is one potential area for improvement.

One clinical trial implemented AI-ECGs in the primary care setting to increase the diagnosis of low LVEF (<50 %). In the study, 120 primary care teams from 45 clinics or hospitals were randomized to access AI-ECG results vs. a control arm (routine care). ECGs were obtained as part of routine care from a total of 22,641 adults without prior heart failure (*N* = 11,573 intervention; N = 11,068 control). The primary outcome was a new diagnosis of low LVEF within 90 days. The trial met this endpoint, demonstrating that the intervention increased the diagnosis of low LVEF in the overall cohort (2.1 % vs. 1.6 %, odds ratio (OR) 1.32 (1.01–1.61), *P* = 0.007) and also found that for patients with positive AI-ECGs, more echocardiograms were obtained in the intervention compared with the control arm (49.6 % vs. 38.1 %, *P* < 0.001), suggesting that the inclusion of AI-ECGs may pick up early and subtle electrocardiographic signs of LV dysfunction that clinicians may miss, but also that significant subsequent testing, at significant expense, was required in order to detect a few more low-EF individuals.^187^ Additional larger multicenter studies could help support the findings.

Attia et al. expanded the use of AI-ECG to single-lead tracings provided by the Apple Watch. They invited patients of the Mayo Clinic to send Apple Watch ECGs to a secure data platform via a phone app. In total, 421 participants had at least one watch-classified sinus rhythm ECG within 30 days of an echocardiogram, among whom 16 (3.8 %) had an LVEF ≤40 %. The AI algorithm detected patients with low LVEF with an AUC of 0.885 (95 % confidence interval 0.823–0.946). Their findings indicate that consumer watch ECGs, acquired in nonclinical environments, can be used to identify patients with cardiac dysfunction, who can often be asymptomatic.^188^ Additional studies are needed to help externally validate these findings and to potentially investigate whether AI analysis of smartwatch ECGs could identify patients who would benefit from further screening for LV dysfunction.

Beyond algorithmic predictions, work is also needed to establish approaches and systems for managing classifications of potential future events. Such approaches must balance the potential promise of SCA prediction minutes to hours beforehand (specifically around the tremendous early resuscitation opportunity), with the false positive implications for both patients and emergency responding systems. Establishing a robust approach to this challenge can help ensure resources are optimally utilized, while also providing an opportunity for algorithms of varying performance profiles to still have a significant impact. For example, establishing low cost, minimally disruptive approaches to handling false positives can enable algorithms with varied sensitivities to provide crucial, potentially life-saving alerts.

## Digital technologies and deployment strategies to bridge gaps in SCA prediction and prevention and improve outcomes from SCA

### Technology for Pre-SCA detection of risk

Though AI/ML models improve prediction of SCD, limitations include accessibility of the required advanced imaging studies and computational algorithms. In addition, we lack studies that demonstrate at what level results may be actionable or whether interventions, such as ICD implantation, might improve outcomes. However, such predictive models may yield populations for whom more active digital surveillance could be targeted.

In an age of easy access to and widespread use of digital technologies, such as smartwatches and smartphones, there has been an increase in access to clinical data such as heart rate measurements and ECGs. Smartwatches have already shown promise in screening for atrial fibrillation (AF) as demonstrated by the Apple Heart Study^189^, the Huawei smartwatch study^190^, the Fitbit Heart Study^191^, and BASEL Wearable Study.^192^ However, the photoplethysmography (PPG) technology used in these smartwatches to identify pulse irregularities not only help us identify AF but could potentially help to detect other forms of frequent ectopic beats. For example, another study by the Apple Heart Study investigators using a smartwatch sought to investigate the prevalence of arrhythmias other than AF in those with a detected irregular pulse. The Apple watch was used to intermittently measure pulse tachograms, and those who received an irregular pulse notification based on the algorithm were notified by the watch and prompted to initiate a telemedicine visit with a physician. Those without urgent symptoms reported during the visit were then mailed an ECG patch to be worn for 7 days and returned by mail. Analysis of the ECG patch results revealed that in patients without AF detected on the ECG patches, 40 % had arrhythmias more significant than short salvos of SVT or pauses, with atrial and ventricular ectopy most commonly detected.^193^

A high burden of PVCs is associated with increased risk for ischemic heart disease^194^, cardiomyopathy, and mortality.^195,196^ The ability of smartwatches to identify such ectopic rhythms could be leveraged to identify more individuals (who may otherwise be asymptomatic and not seek medical attention) who may benefit from further cardiac workup. Early identification of such individuals could prevent the worsening of underlying cardiomyopathies that may result in SCD. Additionally, PVCs have also been shown to increase the risk for sustained VT in patients with myocardial scar.^197^

Additionally, recent studies have sought to analyze whether smart watch ECGs can be used to screen for non-arrhythmic abnormalities, such as ST-segment changes, which might identify acute coronary syndrome (ACS) that might precede SCD. However, single-lead recordings have low sensitivity for detecting ACS (e.g., 34 % in one Apple Watch study).^198^ Thus, the utility of single-lead smartwatch ECGs in the out-of-hospital analysis of chest pain is limited. Multiple lead recordings that might improve sensitivity could utilize wearables paired with wireless electrodes, multiple wearables, or with a smartphone capable of functioning as a second lead. Varying smartwatches’ electrode placement on the body could also yield multiple-lead ECG recordings. One study obtained 4-lead ECGs using the Apple Watch on patients with or without CVD who presented to outpatient offices or the ED by varying watch placement on the body with the index finger on the crown of the watch at all positions. 12-lead ECGs obtained on the same patients were used for confirmation. The study found that the watch-based 4-lead ECGs resulted in sensitivity and specificity of 77 % and 92 % respectively for detecting ST/T abnormalities, marking an improvement from the single wrist lead ECG.^199^ Similarly, another study obtained 9-lead ECGs using the Apple Watch on 100 patients (54 patients with ST-elevation myocardial infarction (STEMI), 27 with non-ST-elevation myocardial infarction (NSTEMI) and 19 healthy), revealed sensitivity and specificity for detecting ST changes of 93 % and 95 %, and for NSTEMI ECG alterations these figures were 94 % and 92 % respectively.^200^

Although the above studies show promising results, there is room for refinement of the PPG and ECG detection technology and algorithms used in smart watches. For example, in the BASEL study, there were notable numbers of inconclusive ECG tracings which lowered the sensitivity and specificity of the devices for AF detection. A manual review of the tracings by a clinician was required to establish a diagnosis in around 25 % of the cases.^192^ Larger studies with extended follow-up periods would be necessary to determine whether such smartwatch-based screening methods for arrhythmia and ischemia could increase early detection of ACS and other CVDs and/or improve clinical outcomes and SCD prevention. Additionally, standardization of data and potential overburdening of healthcare systems due to false alarms are also issues that will need to be addressed.

### Gaps in the chain of survival from cardiac arrest: potential role of digital technologies

Despite much work on the identification of individuals at risk for SCD, we are currently protecting with ICDs only a minority of patients at high risk for SCA. Outcomes after OHCA remain poor. While most OHCA victims do not survive, average mortality figures fail to convey that survival can vary up to 5-fold across communities. Such a large variance highlights that resuscitation outcomes are amenable to intervention and can be materially affected by systems that decrease the time from collapse to resuscitation.^201–203^ An analysis of OHCA registries from 12 countries showed an overall rate of survival to hospital discharge ranging only 6 to 22 %, and survival with cerebral performance category of 1 or 2 (i.e., good neurological outcomes) ranging from 2 to 20 %.^9,204^ CARES data show that in the US, survival to hospital discharge is approximately 10 % overall. Approximately 71 % of SCAs occur at home and approximately half are unwitnessed. Unwitnessed SCA carries an even worse prognosis than observed SCA. Increased automated detection of otherwise unwitnessed SCA could potentially accelerate activation of EMS, community CPR and automatic external defibrillator (AED) resources, potentially improving outcomes for these events that carry the worst prognosis.

**Fig. 3.**
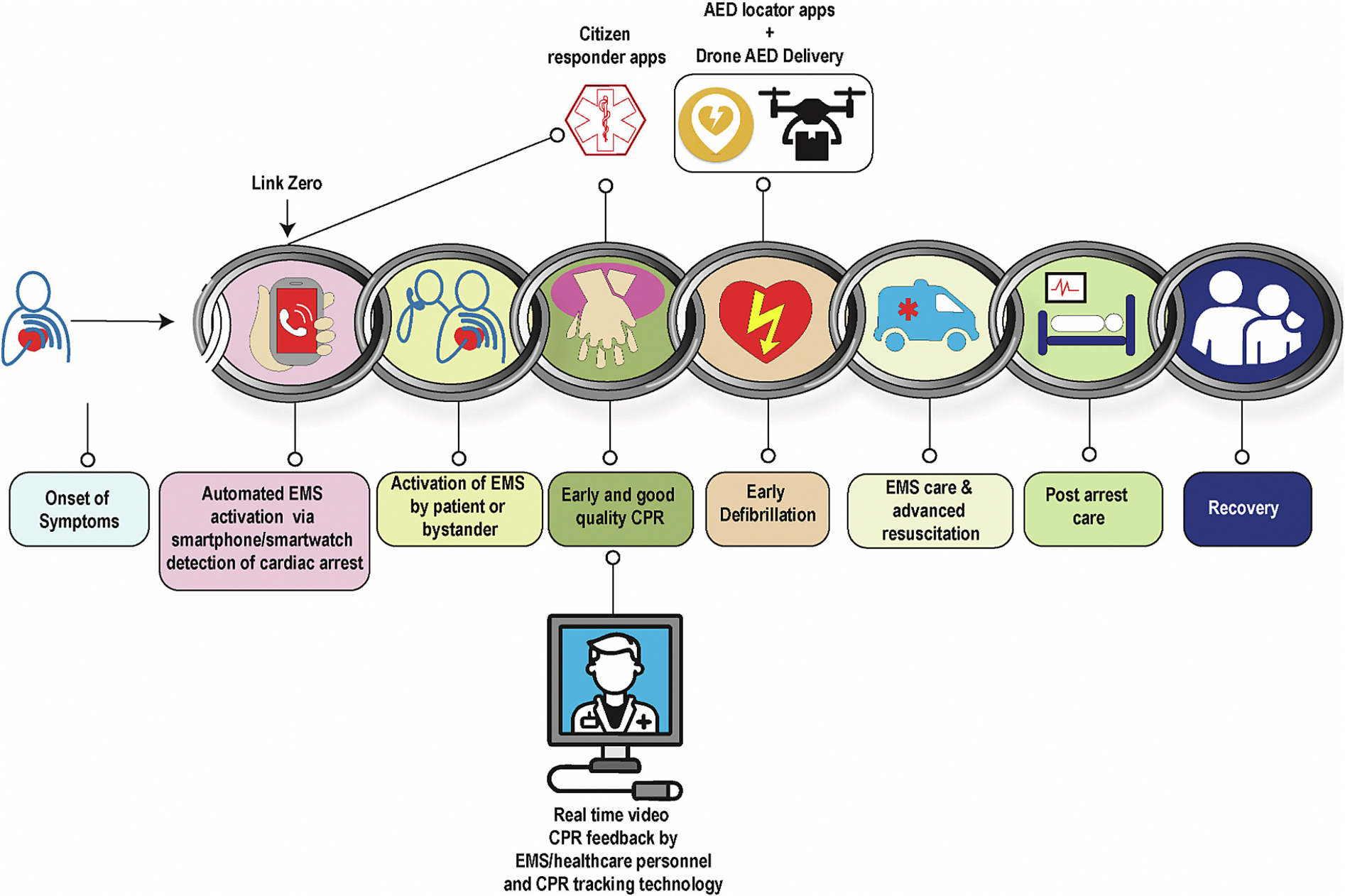
Potential Roles for Technology and AI in Adding to and Strengthening the Chain of Survival from OHCAs.

The six links in the American Heart Association chain of survival from cardiac arrest are: activation of EMS, early CPR, early defibrillation, rapid delivery of EMS care, post-resuscitative care, and recovery.^205^ Technology can play a vital role in strengthening crucial links in this process, and could also play a role in creating an even earlier link focused on acute detection of SCA. Fig. 3 shows the potential role for technology in the survival chain, and these steps are detailed in the text below.

### Technology to improve acute detection of SCA and activation of EMS

Activation of EMS usually tends to be the weakest link in the chain of survival. As mentioned above, warning symptoms precede SCA in more than half of cases. However, as shown in the Oregon SUDS study, only a small minority of patients with warning symptoms call EMS. Public education is needed to teach people how to recognize worrisome symptoms early and then call 911.^139^ However, this weak link of human-dependent EMS activation may benefit from the use of technology that can automatically detect SCA and alert EMS appropriately.

Several wearable and non-wearable sensors could be applied to SCA detection. Some of these sensors include: PPG used to detect pulse, oxygen saturation sensors, fall detection sensors, and ECG. These technologies could be leveraged, preferably in combination, for acute detection of OHCA. ECG or PPG recorders are being developed for use in various wearable and non-wearable forms, such as in smart watches, smart rings, clothing, smart bed sheets, steering wheels, or shopping cart handles.^206–209^ In an effort to automate EMS activation in the Netherlands, the HEART-SAFE (Home Emergency Alerting and Response Technology – Survive A Fatal Event) consortium seeks to develop and implement a technical solution to reliably detect OHCA based on sensor signals, and to automatically alert EMS dispatch centers and nearby CPR volunteers. They aim to use a ML approach to identify patterns related to SCA in data from smartwatches, such as absence of pulsatile blood flow, fall detection, and absence of movement.^210^ Another group in the Netherlands has also proposed a similar model.^211^ The recently released Google Pixel Watch has an algorithm to detect loss of pulse, and can automatically alert EMS.^212^ The algorithm utilizes a multi-step process including continuous heart rate monitoring using its heart rate sensor, then PPG to look for additional signs of a pulse, and motion sensor checks for movements. An AI-based algorithm combines this information to confirm a loss-of-pulse event. An audio alarm and countdown begin, and if no response is detected, the smartwatch automatically places a call to EMS and shares a message indicating that no pulse is detected, along with the location.^213^ While this type of algorithm holds great promise for the early detection of SCA and rapid deployment of EMS, the sensitivity of this algorithm has not yet been tested in a real-world setting.

Agonal breathing is present in approximately half of SCA to which EMS responds.^214^ A group of researchers and physicians from the University of Washington developed an agonal breathing-based ML algorithm to detect SCA. The model was developed using audio of agonal breathing from 911 calls of verified SCA cases. In a proof of principle demonstration in controlled settings, the resulting model had an AUC of 0.9993 for the identification of agonal breathing when implemented in smart devices such as the Amazon Echo and iPhone.^215^ Such algorithms could theoretically be deployed to any smart assistant capable devices, such as smartphones and smart-speakers, to help identify peri-arrest states and allow earlier and increased EMS activation. One major advantage of algorithms based on agonal breathing is that the devices with such audio-based algorithms do not need to be in direct contact with the user; a downside is that only approximately half of SCA contains agonal breathing.^214^ The impressive specificity of such algorithms could help address the challenge of high false positive calls to EMS centers, as has occurred with single-feature (e.g., fall only) detection. Combining agonal breathing data from non-wearable sensors with loss of pulse detection algorithms from wearable devices could result in models with increased versatility and high predictive accuracy, while providing some coverage while watches are being charged during sleep.

Implanted cardiac electronic devices could also provide high-fidelity detection of SCA, as they may be connected to patients’ smartphones which can connect to EMS. Devices such as implantable loop recorders (ILRs) and pacemakers can detect sustained VT/VF but currently do not have connections to EMS. Similarly, there is currently no connectivity to EMS for detection of asystole in ILRs or wearable cardioverter-defibrillators, nor for ICD events during which all shocks are delivered with no success. These are perhaps the lowest-hanging fruit for development, especially since patients with these devices are generally at higher cardiovascular risk than in the general population.

### Accelerating care deployment for SCA: improving bystander CPR and AED use

Patients with a bystander or EMS-witnessed arrest are more than 3 times as likely to survive their event compared with those with unwitnessed arrests.^139,205^ Timely bystander CPR and AED use has been shown to improve survival after SCA by shortening response time to these life-saving treatments. In the United States CARES registry, in 2023 the median response time by first responders was 6.2 min, compared to 7.6 min by EMS.^205^ Besides facilitating detection of the acute event, alerting and dispatch of nearby CPR providers about the SCA occurrence, as well as nearby AED locations, have also been associated with improved survival.^216^

Quality of CPR is known to affect SCA survival.^217^ However, prehospital CPR either by laypersons or professional responders is often suboptimal.^218^ Technology to improve CPR quality by laypersons could be in the form of real time video feedback from healthcare professionals during the event. Human and mannikin studies have shown that improved compression depth and rates resulted from feedback. However, these same studies did not show any improvement in neurological outcome.^219^ Efforts to improve SCA survival in Singapore took CPR quality feedback one step further by providing to registered first responders a CPR card that could be placed on the patient’s chest during compressions that would give real time feedback to optimize compression depth.^220^

However, improving CPR quality may require a more multimodal and patient-tailored approach than was previously thought. Studies have suggested that standard compression hand positions and rates may not be suitable for every patient and may actually compress the LV outflow tract in a large proportion of SCA patients, leading to suboptimal resuscitation.^221–223^ There exist real-time measures that can be obtained which can guide quality of CPR. Some of these metrics include end-tidal CO2 monitoring (an imperfect surrogate for cardiac output) and diastolic blood pressure. Studies in pediatric patients have shown a correlation between higher diastolic pressures and improved survival from SCA in centers in which hemodynamic directed CPR was practiced.^224^ Improved technology could be developed to monitor patients’ physiological parameters such as diastolic blood pressure and end-tidal CO2, and to provide real-time feedback to rescuers on how to tailor compressions. Such technology could also be incorporated into mechanical compression devices to improve mechanical CPR.^9^

Another way to improve the chain of survival is to augment access to AEDs and to educate the public on their appropriate use. Public AEDs are being increasingly deployed, yet bystander defibrillation occurs only in 2 % to 12 % of all OHCAs.^205,225–227^ Interventions to increase access to publicly available AEDs in the Netherlands have already helped improve AED attachment rates and subsequent survival from shockable rhythms.^228^ In addition, a Swedish-based study assessed the drone-based delivery of AEDs to 30 m or less from the site of OHCA.^229^ In most cases in which a drone was deployed, AEDs were delivered within a median time of 3 min before EMS arrival, allowing for AED attachment in 6 out of 18 cases (33 %) in which AED attachment was warranted. The study suggests that AED-equipped drones could complement ambulances, particularly in light of worsening ambulance response times reported in recent studies.^230^ This strategy could also complement stationary AEDs, as there often is spotty coverage in residential areas where the majority of OHCA take place,^231^ and this strategy also tackles the challenging issues of maintenance and misplacement of AEDs. Another potential strategy is to redesign the AED^232^ to make it significantly more affordable and user-friendly so that they can be deployed in many more households, which is where 83 % of OHCAs occur.^205^

Dispatch of community responders also has shown improvements in rates of bystander CPR, AED use and survival. A prospective observational study in Denmark dispatched citizen responders to the scenes of OHCAs using a smartphone app.^233^ The app was also linked to a map of the Danish AED network, making it easy for responders to identify the locations of the nearest AEDs. Citizen responders were alerted in 819 instances of suspected OHCAs, out of which 438 cases (54 %) were confirmed OHCA incidents that met the study’s inclusion criteria. In 42 % (184 out of 438) of the confirmed OHCA cases, at least one citizen responder arrived before the arrival of EMS. The odds for bystander CPR increased (odds ratio: 1.76; 95 % confidence interval: 1.07 to 2.91; *p* = 0.027) and the odds for bystander defibrillation more than tripled (odds ratio: 3.73; 95 % confidence interval: 2.04 to 6.84; *p* < 0.001) compared with OHCAs in which citizen responders arrived after EMS.^233^ A large clinical trial to evaluate the effects of this methodology on survival from OHCA is ongoing. Another retrospective observational study was performed using data collected from 5 European sites participating in a collaborative research network.^216^ By means of a website or a smartphone application, volunteer responders registered and consented to CPR training and to be located and dispatched in case of an OHCA nearby. The emergency medical communication center, triggered by dispatchers in response to an emergency call concerning a suspected case of OHCA, alerted nearby responders. Compared with no system activation, activation of this volunteer response system was associated with a higher chance of bystander CPR, bystander defibrillation, and, importantly, 30-day survival.^216^

Similarly, in certain regions of the US, a mobile app called PulsePoint is connected to EMS and alerts registered responders of nearby cardiac arrests. The app also has an AED map to facilitate defibrillation. The app has over 1 million active users.^234^ Whether initiatives such as these can reduce first responder arrival times, improve the chance that arrests are witnessed, and improve survival will be important to document. Activation of the chain of response still hinges on the initial call being made to EMS, omitting from documentation the large proportion of SCA patients who do not or are unable to call EMS. Employing digital technologies to aid in acute detection of SCA with automated connection to EMS will require identification and harmonization of standards for identifying SCA, facilitating connections to EMS, education of the public to use and activate the technologies, and addressing barriers to access and use.

Apart from improving access to AEDs, novel defibrillation techniques have also been developed to improve first shock success. A randomized controlled trial showed an improvement in survival with double sequential defibrillation, in which two separate defibrillators are used on the same patient delivering rapid sequential shocks through both standard and anterior–posterior pad positions.^235^ Another study implemented signal filtering techniques into AEDs, allowing the analysis of ECG signals without pausing chest compressions and resulting in fewer pauses in chest compressions.^236^

### Quality control and regulatory challenges

Emerging AI-enabled technologies have the potential to revolutionize medical practice and improve the prediction of SCA. But these new big data multifaceted approaches also bring challenges including privacy risks and data-sharing barriers that need to be overcome.^237^ It is important to apply AI SCD predicting solutions with consideration to the patient’s needs, trust, and safety as well as clinical workflow and ethical implications.^237^ Crucial steps are required to ensure these risks are addressed in a patient-centered, collaborative regulatory approach with transparency, accountability, and health equity advanced by the Food and Drug Administration (FDA).^237^

### Ongoing efforts and registries

There already are multifaceted national efforts to improve SCA survival.^238^ The American Heart Association’s Get With The Guidelines-Resuscitation (GTWG-R), previously known as the National Registry of Cardiopulmonary Resuscitation is a large, multicenter observational registry of in-hospital cardiac arrests. In 2004, the U.S. Centers for Disease Control and Prevention (CDC) established the Cardiac Arrest Registry to Enhance Survival (CARES) in collaboration with the Emory University School of Medicine. CARES was developed to help communities determine standard outcome measures for OHCAs, by linking the three sources of information: 911 dispatch centers, EMS providers, and receiving hospitals. The CARES registry allows EMS systems to compare their performance with aggregate national measures and benchmarks. The registry has expanded to several states, representing a catchment of approximately 175 million people or 53 % of the US population. Information on neurological outcomes after SCA is also included in the registry.

Additionally, The Resuscitation Academy Foundation (RAF) was founded in 2008 to publicize Seattle/King County’s lessons on optimizing an EMS system’s chain of survival. EMS leaders from around the world gather in Seattle twice a year to exchange ideas on how to save more lives. RAF created the Lighthouse program, which requires organizations to meet high standards in several areas to achieve “Lighthouse” designation. These areas include registry use, CPR training, regular feedback to responders, and commitments to improve and help other communities. Organizations in various states such as Maryland and Kentucky have achieved Lighthouse designation and have shown improvements in their CPR and Utstein survival metrics.^239^ For example, Jessamine County in Maryland has shown an increase in bystander CPR with dispatcher assistance from 9.4 % in 2019 to 36.6 % in 2022, and also showed an improvement in Utstein survival from 12.5 % to 25 % in the same period. Similarly, in Dane county, Wisconsin, with the help of telecommunicator-assisted CPR by the 911 communications center, bystander CPR rates increased from 40.2 % in 2021 to 62.8 % in 2022.^239^

By showing the important effect of bystander CPR on SCA survival, the CARES registry also highlights the possible importance of youth CPR. As of December 31, 2020, 39 out of 50 states and the District of Columbia mandated CPR and AED training for high school students.^239^ In states with CPR/AED training laws compared to those without such laws, a greater percentage of patients who experienced OHCAs received bystander CPR, regardless of gender, race, ethnicity, arrest witness status (witnessed/unwitnessed), location of arrest (public/residential), or initial presenting cardiac rhythm (shockable/non shockable).^239^

Registries such as CARES and organizations such as the RAF are important because they serve as a repository for various SCA metrics such as EMS response times, rates of bystander CPR, survival rates etc., and give communities across the country a standard to strive towards. CARES data already show significant regional variability in SCA survival across participating communities. Closely examining each link in the chain of survival in poorly performing communities (compared with overall national benchmarks) enables each community to make the necessary systemic interventions to improve their outcomes. Organizations such as PulsePoint that mobilize citizen responders also are working to improve bystander CPR and AED use. It is also hoped that the recently passed HEARTS Act will increase attention to school CPR education and AED access.^240^ The Heart Rhythm Society has launched a task force to help facilitate implementation of the HEARTS Act.

## Call to action/conclusion

Despite much work on SCD risk prediction, we are currently protecting only a minority of patients at high risk for SCA. SCA remains a major public health problem with overall survival of only 10 %. Thus, the medical community is tasked with the challenge to improve prediction and prevention strategies, as well as to overcome barriers to implementing and accelerating the detection of long- and near-term risk and the acute SCA event itself, potentially through AI-assisted algorithms and integration with EHR, ECG, digital wearable and non-wearable technologies (Central Illustration). While various studies have shown that ML models are able to predict SCA with high accuracies, further research efforts should be directed towards externally validating such models in clinical settings to truly assess whether they can help improve SCA prediction. A report from a Lancet commission noted that research funding has been focused on advanced resuscitation and post-arrest care, but that the largest impact on survival is earlier in the chain of survival, at activation of emergency response, high quality CPR and defibrillation,^9^ and even earlier than these first links, at detection of the acute SCA event. We call for medical technology corporations including digital health and implantable device industries, academic researchers, professional and patient advocacy societies, and our communities to collaborate to improve response to and outcomes of SCA.

## Figures and Tables

**Table 1:** Etiologies of SCD in Western Countries^13^.

Etiology	Percentage

Coronary Artery Disease	~70 % (lower in Women and African-Americans)
Cardiomyopathies	10–15 %
Inherited Arrhythmia Syndromes	1–2 %
Valvular Heart Disease	1–5%
No cardiac abnormality on autopsy	~30–40 %*

* In persons 1–49 years old per some autopsy series^17,18^.

**Table 2:** Clinical trials for secondary prevention of SCD.

Study	No.	Randomization	Population	Mean Follow-up (months)	Mortality (HR) and *p*-value

AVID^20^ (AVID investigators 1997)	1016	Antiarrhythmic medications (97 % amiodarone, 3 % sotalol) vs ICD	Survived VT/VF/cardiac arrest; VT with syncope; VT with LVEF ≤40 %	18	0.66 (*p* < 0.02)
CASH^21^ (Kuck et al., 2000)	288	Antiarrhythmic medications propafenone (withdrawn early), metoprolol, or amiodarone vs ICD	Survived VT/VF/cardiac arrest	57	0.82 (p = 0.08)
CIDS^22^ (Connolly et al., 2000)	659	Amiodarone vs ICD	Survived VT/VF/cardiac arrest; VT with syncope; VT with LVEF ≤35 % and cycle length ≤ 400 ms	35	Reduction in death from arrhythmia 0.85 (*p* = 0.09)
DEBUT^23^ (Nademanee et al., 2003)	86	beta-Blocker vs ICD	Survived VT/VF/cardiac arrest; no structural abnormalities	36	All deaths occurred in beta-blocker group (p = 0.02 at 3 year follow up)
MAVERIC^24^ (Lau et al., 2004)	214	Electrophysiologically guided therapy (antiarrhythmic, revascularization, or ICD) vs amiodarone	Survived VT/VF/cardiac arrest	60 (median)	0.54 (p = 0.04)

AVID - Antiarrhythmics versus Implantable Defibrillators. CASH - the Cardiac Arrest Study Hamburg. CIDS - Canadian implantable defibrillator study. DEBUT - Defibrillator Versus beta-Blockers for Unexplained Death in Thailand. ICD = Implantable cardioverter-defibrillator. LVEF = left ventricular ejection fraction. MAVERIC - The Midlands Trial of Empirical Amiodarone versus Electrophysiology-guided Interventions and Implantable Cardioverter-defibrillators. VT = ventricular tachycardia. VF = ventricular fibrillation.

**Table 3:** Summary of recommendations and clinical trials for prevention of SCD in ischemic heart disease.

Condition	Primary prevention (Strength of Recommendation)			Secondary prevention (Strength of Recommendation)		

Ischemic Heart Disease	Ischemic heart disease at least 40 days post-MI and at least 90 days after coronary revascularization with LVEF of 35 % or less and NYHA Class II or III symptoms on chronic GDMT, who have reasonable expectation of meaningful survival for more than 1 year (I)^59^ In selected patients at least 40 days post-MI and at least 90 days after coronary revascularization with LVEF of 30 % or less, NYHA Class I symptoms while receiving GDMT, who have a reasonable expectation of meaningful survival for more than 1 year (I)^59^			Patients with ischemic heart disease who either survive SCA or experience hemodynamically unstable VT or stable sustained VT not due to reversible causes and have a reasonable expectation of meaningful survival for more than 1 year (I)^58^		

Study	No.	Randomization	Population	Timing of ICD	Mean Follow-up (months)	Mortality (HR) and p-value

MADIT^60^ (Moss et al., 1996)	196	Antiarrhythmic therapy (74 % amiodarone) vs ICD	Prior MI; LVEF ≤35 %; asymptomatic NSVT; NYHA class I-III; inducible VT refractory to IV procainamide on EPS	>3 weeks post-MI >3 months post revasc	27	0.46 (*p* = 0.009)
MUSTT^61^ (Buxton et al., 1999)	704	Electrophysiologically guided therapy (antiarrhythmic drug or ICD) vs conventional therapy	Prior MI; LVEF ≤40 %; CAD; NSVT; inducible VT on EPS	≥4 days post-MI or revascularization (Median time from MI to randomization: 39 months)	39 (median)	0.40 (p < 0.001)
MADIT-II^62^ (Moss et al., 2002)	1232	Conventional therapy vs ICD	Prior MI; LVEF ≤30 %	>1 month post MI >3 months post revasc	20	0.69 (*p* = 0.016)
SCFHeFT^55^ (Bardy et al., 2005)	2521	Conventional therapy vs amiodarone vs ICD	NYHA class II/III CHF (ischemic and nonischemic); LVEF ≤35 %	≥6 months post-MI (75 % of patients)	45.5 (median)	0.79 (*p* = 0.05)
DINAMIT^64^ (Hohnloser et al., 2004)	674	Conventional therapy vs ICD	Recent MI, LVEF ≤35 %; decreased HRV or avg. HR ≥ 80 bpm	6–40 days post-MI	39	1.08 (*p* = 0.66)
IRIS^63^ (Steinbeck et al., 2009)	898	Conventional therapy vs ICD	Recent MI, LVEF≤40 %; HR > 90 bpm or NSVT>150 bpm	3–31 days post-MI	37	1.04 (*p* = 0.78)

Avg = average. Bpm = beats per minute. CHF = congestive heart failure. DINAMIT - The Defibrillator in Acute Myocardial Infarction Trial. EPS = electrophysiological study. GDMT = guideline directed medical therapy. HR = heart rate. HRV = heart rate variability. ICD = implantable cardioverter-defibrillator. IRIS - Immediate Risk Stratification Improves Survival trial. IV = intravenous. LVEF = left ventricular ejection fraction. MADIT - The Multicenter Automatic Defibrillator Implantation Trial. MI = myocardial infarction. MUSTT - Multicenter UnSustained Tachycardia Trial. NSVT = non-sustained ventricular tachycardia. NYHA = New York Heart Association. Revasc = revascularization. SCA = sudden cardiac arrest. SCDHeFT - The Sudden Cardiac Death in Heart Failure Trial. VT = ventricular tachycardia.

**Table 4:** Summary of recommendations and clinical trials for prevention of SCD in nonischemic cardiomyopathy.

Condition	Primary prevention (Strength of Recommendation)			Secondary prevention (Strength of Recommendation)	

Non-ischemic Cardiomyopathy (NICM)	NICM with LVEF of 35 % or less and NYHA Class II or III symptoms on chronic GDMT, who have reasonable expectation of meaningful survival for more than 1 year (I)^59^			Patients with NICM who either survive SCA due to VT/VF or experience hemodynamically unstable VT or stable sustained VT not due to reversible causes, an ICD is recommended if meaningful survival greater than 1 year is expected (I)^58^	

Study	No.	Randomization	Population	Mean Follow-up (months)	Mortality (HR) and p-value

CAT^71^ (Bänsch et al.)	104	Conventional therapy vs ICD	NYHA class II/III, NICM; LVEF ≤30 %; asymptomatic NSVT	66	No reduction in total mortality with ICD therapy (*P* = 0.55)*
AMIOVIRT^72^ (Strickberger et al.)	103	Amiodarone vs ICD	NYHA class I-III, NICM; LVEF ≤35 %; asymptomatic NSVT	36	No reduction in total mortality with ICD therapy (*P* = 0.80)*
DEFINITE^68^ (Kadish et al.)	458	Conventional therapy vs ICD	NICM; LVEF <36 %; NSVT or PVCs	29	0.65 (*p* = 0.08)
SCDHeFT^55^ (Bardy et al.)	2521	Conventional therapy vs amiodarone vs ICD	NYHA class II/III CHF (ischemic and nonischemic); LVEF ≤35 %	45.5 (median)	0.73 (*p* = 0.06)
DANISH^69^ (Køber et al.)	1116	Usual clinical care vs ICD	NYHA class II/III, NICM; LVEF ≤35 %	67.6 (median)	0.87 (*p* = 0.28) SCD mortality 0.50 (*p* = 0.005)

AMIOVIRT - The Amiodarone versus implantable cardioverter-defibrillator trial. CAT - The Cardiomyopathy Trial. DANISH - The Danish Study to Assess the Efficacy of ICDs in Patients with Non-ischemic Systolic Heart Failure on Mortality trial. DEFINITE - The Defibrillators in Non-Ischemic Cardiomyopathy Treatment Evaluation. GDMT = guideline directed medical therapy. ICD = implantable cardioverter defibrillator. LVEF = left ventricular ejection fraction. NICM = Nonischemic cardiomyopathy. NSVT = non-sustained ventricular tachycardia. NYHA = New York Heart Association. PVC = premature ventricular contraction. SCA = sudden cardiac arrest. SCD = sudden cardiac death. SCDHeFT - The Sudden Cardiac Death in Heart Failure Trial. VF = ventricular fibrillation. VT = ventricular tachycardia.

* Hazard ratios and confidence intervals not reported.

**Table 5:** ICD recommendations for various etiologies of SCD.

Condition	Primary prevention (Strength of Recommendation)	Secondary prevention (Strength of Recommendation)

Arrhythmogenic Right Ventricular Cardiomyopathy (ARVC)	ARVC and syncope suspected to be due to VA (IIa)^73^ ARVC and three major, two major and two minor, or one major and four minor risk factors for VA (IIa)^73^	ARVC with history of sustained VT, not hemodynamically tolerated (I)^73^ Patients with ARVC and resuscitated SCA ICD is recommended if meaningful survival greater than 1 year expected (I)^73^ ARVC with hemodynamically tolerated sustained VT (IIa)^73^
Arrhythmogenic Left Ventricular Cardiomyopathy (ALVC)	In individuals with ACM with LVEF ≤35 % and NYHA class II-III symptoms and an expected meaningful survival >1 year, an ICD is recommended (I)^73^ In individuals with ACM with LVEF ≤35 % and NYHA class I symptoms and an expected meaningful survival >1 year, an ICD is reasonable (IIa)^73^	In individuals with ACM and hemodynamically tolerated VT, an ICD is recommended (I)^73^
LMNA Cardiomyopathy	In patients with NICM due to a Lamin A/C mutation who have 2 or more risk factors (NSVT, LVEF <45 %, nonmissense mutation, and male sex), an ICD can be beneficial if meaningful survival of greater than 1 year is expected (IIa)^58^	Patients who survive SCA due to VT/VF or experience hemodynamically unstable VT or stable sustained VT not due to reversible causes, an ICD is recommended if meaningful survival greater than 1 year is expected(I)^58^
Hypertrophic Cardiomyopathy (HCM)	Adult patients with HCM with ≥1 of the following major risk factors; sudden death judged definitively or likely attributable to HCM in ≥1 first-degree or close relatives who are ≤50 years of age, massive LVH ≥30 mm in any LV segment; ≥1 recent episodes of syncope suspected by clinical history to be arrhythmic, LV apical aneurysm with transmural scar or LGE on CMR and LVEF <50 % (IIa)^99^ Consideration of ICD in select adult patients with HCM and without major SCD risk factors if the patients are noted to have extensive LGE by CMR or NSVT present on ambulatory monitoring (IIb)^99^	HCM and previous documented cardiac arrest or sustained VT (I)^99^
Long QT Syndrome (LQTS)	In high-risk patients with long QT syndrome unresponsive or intolerant to beta blockers, intensification of therapy with additional measures such as an ICD (I)^58^ In asymptomatic patients with long QT syndrome and a resting QTc greater than 500 ms while receiving a beta blocker, intensification of therapy with medications, left cardiac sympathetic denervation or an ICD may be considered (IIb)^58^	Patients with known LQTS resuscitated from cardiac arrest (I)^58^
Brugada Syndrome (BrS)	In patients with BrS with spontaneous type 1 Brugada ECG and a history of syncope judged to be caused by VA, an ICD is recommended (I)^58^	In patients with BrS with spontaneous type 1 Brugada ECG and a history of sustained VA, an ICD is recommended (I)^58^ Patients with known BrS and history of cardiac arrest, if meaningful survival of greater than 1 year is expected (I)^58^
Catecholaminergic polymorphic ventricular tachycardia (CPVT)	In patients with CPVT and syncope, while receiving adequate or maximally tolerated beta blocker, treatment intensification with either combination medication therapy (eg, beta blocker, flecainide), left cardiac sympathetic denervation, and/or an ICD is recommended (I)^58^	Patients with known CPVT and recurrent sustained VT while receiving adequate or maximally tolerated beta blocker, treatment intensification with either combination medication therapy (eg, beta blocker, flecainide), left cardiac sympathetic denervation, and/or an ICD is recommended (I) Patients with known CPVT and history of cardiac arrest, if meaningful survival of greater than 1 year is expected (I)^58^
Short QT Syndrome (SQTS)		In patients with SQTS and a history of sustained VA, ICD is recommended (I)^58^ Patients with known SQTS and history of cardiac arrest, if meaningful survival of greater than 1 year is expected (I)^58^

CMR = cardiac magnetic resonance imaging. ECG = electrocardiogram. ICD = implantable cardioverter defibrillator. LGE = late gadolinium enhancement. LV = left ventricle. LVEF = left ventricular ejection fraction. LVH = left ventricular hypertrophy. NICM = Nonischemic cardiomyopathy. NSVT = non-sustained ventricular tachycardia. SCA = sudden cardiac arrest. VA = ventricular arrhythmia. VF = ventricular fibrillation. VT = ventricular tachycardia.

**Table 6:** Summary of ML models for long term prediction of SCD and ventricular arrhythmias.

Study	Source Population for Model Training Data	Factors Incorporated into Prediction Model(s)	Primary Outcome(s)	AUC	External Validation

Improved prediction of SCD in patients with HF through ECG digital processing.^143^	2559 patients discharged after hospitalized for acute decompensated HF in tertiary care centers in Tokyo.	**AI-ECG** + LVEF ≤35 % + NYHA Class II and III vs. LVEF ≤35 % + NYHA Class II and III	Composite SCD events (SCD and ICD activation for shock or ATP) over 3 years.	0.66 for ML model 0.59 for standard guidelines	No
Arrhythmic SD survival prediction using DL analysis of scarring in the heart.^153^	156 patients with ICM enrolled in the Left Ventricle Structural Predictors of Sudden Cardiac Death prospective observational study.	cMRI images +22 clinical covariates.	Estimated probability of SCD in a 10 year period.	0.87	Yes
Substrate Spatial Complexity Analysis for the Prediction of VA in Patients With ICM.^154^	167 patients with ICM from the left ventricular structural predictors of SCD registry, who met criteria for primary prevention ICD placement by LVEF ≤35 % and had no contraindications to cMRI.	Grayscale cMRI data used to generate substrate spatial complexity profiles for each patient, which were used to train ML model.	Predicting appropriate ICD shock for sustained VT, VF or SCD presumed due to VA over 5 years of follow-up.	0.72	No
CinE caRdiac magneTic resonAnce to predIct veNTricular arrhYthmia (CERTAINTY).^155^	350 patients with a history of ischemic or nonischemic left ventricular systolic dysfunction with stable NYHA Class II to III HF symptoms for ≥3 months on optimal pharmacotherapy and an LVEF≤35 % who received an ICD for primary prevention of SCD.	cine cMRI images to train ML model and derive a risk score.	Predicting VA (appropriate ATP without shock or appropriate ICD shock) over a median follow up period of 7.1 years.	0.69	No
ML-based risk model using 123I-MIBG to differentially predict modes of cardiac death in HF.^156^	526 patients with CHF with a mean LVEF of 38 % ± 14 % who were assessed by 123I-MIBG imaging at one of four participating hospitals.	Cardiac 123I-MIBG indices (late HMR and washout rate), age, gender, NYHA functional class, eGFR, LVEF, hemoglobin, BNP/NTProBNP grade, hemodialysis, ischemic etiology, hypertension, and diabetes mellitus.	Arrhythmic events including SCD and appropriate ICD or CRT-D shock or ATP. Other primary endpoints were HF death or survival. Patients were followed for a mean of 30 ± 20 months.	0.8 for arrhythmic event 0.92 for heart failure death	No
Baseline and Dynamic Risk Predictors of Appropriate ICD Therapy.^157^	382 patients with LVEF ≤35 % who underwent cMRI before primary prevention ICD insertion.	cMRI imaging indices, demographics, comorbidities, medications, lab values, electrophysiologic parameters, enrollment LVEF, serial LVEFs, CHF hospitalizations, biomarkers of inflammation such as IL-6.	Appropriate ICD shock for VT above the programmed rate cutoff (generally 180 bpm) or VF or definite or suspected SCD.	0.88	No

123 l-MIBG = 123I-metaiodobenzylguanidine. AI = artificial intelligence. ATP = anti-tachycardia pacing. BNP = brain natriuretic peptide. CHF = congestive heart failure. cMRI = cardiac magnetic resonance imaging. CRT-D = cardiac resynchronization therapy defibrillator. DL = deep learning. eGFR = estimated glomerular filtration rate. ECG = electrocardiogram. HF = heart failure. HMR = heart-to-mediastinum ratio. ICD = implantable cardioverter defibrillator. ICM = ischemic cardiomyopathy. IL-6 = interleukin-6. LVEF = left ventricular ejection fraction. ML = machine learning. NTProBNP = N-terminal pro-b-type natriuretic peptide. NYHA = New York Heart Association. SCD = sudden cardiac death. SD = sudden death. VA = ventricular arrhythmia. VF = ventricular fibrillation. VT = ventricular tachycardia.

**Table 7:** Summary of ML models for prediction of SCD and ventricular arrhythmias in patients with HCM.

Study	Source Population for Model Training Data	Factors Incorporated into Prediction Model(s)	Primary Outcome(s)	AUC	External Validation

A ML-based risk stratification model for VT and HF in HCM.^159^	2302 patients (Italy) primarily diagnosed with HCM or had an HCM-diagnosed relative.	Disease related events such as abnormal test results (ex. Abnormal Holter), medical procedures (ex. Pacemaker or ICD implant), death (ex. SCD, all cause death) and life saving treatment (ex. Appropriate ICD shock, heart transplant).	Estimating risk for SCD, VT, HF, appropriate ICD shock over a 5 year period.	0.7 for SCD	No
Assessment of ventricular tachyarrhythmia in patients with HCM with ML-based texture analysis of LGE cMRI.^160^	64 patients with HCM who underwent cMRI.	Quantitative textural features extracted via manually placed regions of interest in areas with high and intermediate signal intensity on LGE cMRI images.	Predicting presence or absence of VT, using 24 h holter obtained within 1 year prior to cMRI image acquisition as a reference to confirm presence or absence of VT.	0.92	No
Identifying Ventricular Arrhythmias and Their Predictors by Applying Machine Learning Methods to Electronic Health Records in Patients With Hypertrophic Cardiomyopathy (HCM-VAr-Risk Model).^161^	711 patients with established HCM	Type of HCM (obstructive vs not), family history of HCM and SCD, history of syncope, NSVT, LVEF, age, BMI, statin use, stress echo parameters such as LVOT gradient etc.	Predicting risk of lethal arrhythmias including sustained VT or VF, confirmed by reviewing ECG, Holter or ICD interrogations over mean follow up of 2.86 years.	0.83	No

BMI = body mass index. cMRI = cardiac magnetic resonance imaging. ECG = electrocardiogram. HCM = hypertrophic cardiomyopathy. HF = heart failure. ICD = implantable cardioverter defibrillator. LGE = late gadolinium enhancement. LVEF = left ventricular ejection fraction. LVOT = left ventricular outflow tract. ML = machine learning. SCD = sudden cardiac death. NSVT = non-sustained ventricular tachycardia. VT = ventricular tachycardia. VF = ventricular fibrillation.

**Table 8:** Summary of ML models for the near term prediction of SCD and ventricular arrhythmias in the hospital setting.

Study	Source Population for Model Training Data	Factors Incorporated into Prediction Model(s)	Primary Outcome(s)	AUC	External Validation

An Algorithm Based on DL for Predicting IHCA.^176^	52,131 patients admitted to two hospitals, 419 of whom had IHCA.	SBP, heart rate, respiratory rate and body temperature.	Predicting IHCA 14 h prior to event.	0.85 (ML model) vs 0.63 (MEWS)	Yes. (AUC 0.837)
ML for early prediction of in-hospital cardiac arrest in patients with ACS.^178^	166 ACS patients who had IHCA.	Age, gender, history of smoking, history of drinking, ACS type, culprit artery, comorbidities and the number of days prior to the occurrence of cardiac arrest. Laboratory features, Killip classification, vital signs, mental status, imaging, ECG, were recorded 24 h preceding cardiac arrest.	Predicting IHCA 24 h prior to event.	0.958 (ML model) vs 0.673 (MEWS)	No
AI algorithm for predicting cardiac arrest using ECG.^179^	25,672 adult patients admitted to two hospitals, some of whom had IHCA.	ECG, age, sex	Predicting IHCA 24 h from ECG	0.91	Yes. (AUC 0.948)
ML-based risk prediction of malignant arrhythmia in hospitalized patients with HF.^181^	2794 hospitalized HF patients.	103 clinical features including LVEF, lab values such as CKMB, D-dimer, antiarrhythmic drug use, presence or absence of ECG features such as LBBB etc.	Predicting malignant arrhythmias (sustained VT or VF) during hospitalization.	0.867	No
Prediction of VT One Hour before Occurrence Using Artificial Neural Networks.^182^	15 patients hospitalized in the CCU	Real time vital signs data and ECG data collected from CCU monitors and used to measure HRV and RRV prior onset of VT.	Predicting VT one hour prior to event.	0.93	No
Prediction of cardiac arrest in critically ill patients presenting to the ED using a ML score incorporating HRV compared with the MEWS.^183^	925 critically ill patients (Patient Acuity Category Scale 1 and 2) in an ED of a tertiary hospital, 43 of whom had IHCA.	HRV generated from 5 min ECG recording, age, vital signs, collected in the ED.	Predicting IHCA within 72 h.	0.781 (ML model) vs 0.680 (MEWS)	No
Prediction of Cardiac Arrest in the ED Based on ML and Sequential Characteristics: Model Development and Retrospective Clinical Validation Study.^184^	214,307 patients who presented to the ED of a tertiary care hospital, 993 of whom had IHCA.	Age, sex, chief complaint, serial vital signs, collected in the ED.	Predicting IHCA during admission.	0.97 (ML model) vs 0.76 (MEWS)	No
Developing neural network models for early detection of cardiac arrest in the ED.^185^	233,763 patients who presented to the ED of a tertiary care hospital, 1097 of whom had IHCA.	Age, sex, vitals and other clinical parameters, collected in the ED.	Predicting IHCA 24 h prior to event.	0.936	No
Prediction of adverse cardiac events in ED patients with chest pain using ML for variable selection.^186^	702 patients with non- traumatic chest pain in the ED of a tertiary hospital.	SBP, HRV, RRV, collected in the ED	Predicting a composite of death, cardiac arrest, sustained VT and hypotension requiring inotropes or IABP, during admission.	0.812 (ML model) vs 0.622 (MEWS)	No

AUC = Area under the receiver operator characteristic curve. ACS = acute coronary syndrome. CCU = coronary care unit. CKMB = creatinine kinase-MB. DL = deep learning. ECG = electrocardiogram. ED = emergency department. HF = heart failure. HRV = heart rate variability. IABP = intra-aortic balloon pump. IHCA = in-hospital cardiac arrest. RRV = respiratory rate variability. IHCA = in-hospital cardiac arrest. LBBB = left bundle branch block. LVEF = left ventricular ejection fraction. MEWS = modified early warning system. ML = machine learning. SBP = systolic blood pressure. VF = ventricular fibrillation. VT = ventricular tachycardia.

## Abbreviation list:

ACM: Arrhythmogenic Cardiomyopathy
ACS: Acute Coronary Syndrome
ACHD: Adult Congenital Heart Disease
AED: Automated External Defibrillator
AF: Atrial Fibrillation
AI: Artificial Intelligence
AI-ECG: Artificial Intelligence Electrocardiogram
ARVC: Arrhythmogenic Right Ventricular Cardiomyopathy
ALVS: Arrhythmogenic Left Ventricular Cardiomyopathy
ATP: Antitachycardia Pacing
AUC: Area Under the Curve
AVID: Antiarrhythmics Versus Implantable Defibrillators
BUN: Blood Urea Nitrogen
BrS: Brugada Syndrome
CAD: Coronary Artery Disease
CARES: Cardiac Arrest Registry to Enhance Survival
CASH: Cardiac Arrest Study Hamburg
CDC: Centers for Disease Control and Prevention
CHF: Congestive Heart Failure
CHD: Congenital Heart Disease
CIDS: Canadian Implantable Defibrillator Study
CI: Confidence Interval
cMRI: Cardiac Magnetic Resonance Imaging
CPR: Cardiopulmonary Resuscitation
CPVT: Catecholaminergic Polymorphic Ventricular Tachycardia
CRT: Cardiac Resynchronization Therapy
CRT-D: Cardiac Resynchronization Therapy with Defibrillator
CVD: Cardiovascular Disease
DANISH: Defibrillator Implantation in Patients with Nonischemic Systolic Heart Failure
DCM: Dilated Cardiomyopathy
DEWS: Deep-Learning-Based Early Warning Score
DINAMIT: Defibrillator in Acute Myocardial Infarction Trial
DL: Deep Learning
DSP: Desmoplakin
ECG: Electrocardiogram
ED: Emergency Department
EF: Ejection Fraction
EMS: Emergency Medical Services
EPS: Electrophysiology Study
FDA: Food and Drug Administration
FLNC: Filamin C
GDMT: Guideline-Directed Medical Therapy
GTWG-R: Get With The Guidelines – Resuscitation
HCM: Hypertrophic Cardiomyopathy
HF: Heart Failure
HFSA: Heart Failure Society of America
HMR: Heart/Mediastinal Ratio
HRS: Heart Rhythm Society
HRV: Heart Rate Variability
HTN: Hypertension
ICD: Implantable Cardioverter Defibrillator
ICM: Ischemic Cardiomyopathy
IHCA: In-Hospital Cardiac Arrest
ILR: Implantable Loop Recorder
I-MIBG: 123I-metaiodobenzylguanidine
IRIS: Immediate Risk Stratification Improves Survival
IVCD: Intraventricular Conduction Delay
LBBB: Left Bundle Branch Block
LGE: Late Gadolinium Enhancement
LMNA: Lamin A/C
LQTS: Long QT Syndrome
LV: Left Ventricle
LVEF: Left Ventricular Ejection Fraction
MADIT: Multicenter Automatic Defibrillator Implantation Trial
MEWS: Modified Early Warning Score
MI: Myocardial Infarction
ML: Machine Learning
MUSTT: Multicenter UnSustained Tachycardia Trial
NICM: Non-Ischemic Cardiomyopathy
NSR: Normal Sinus Rhythm
NSTEMI: Non-ST-elevation Myocardial Infarction
NSVT: Non-Sustained Ventricular Tachycardia
NYHA: New York Heart Association
OHCA: Out-of-Hospital Cardiac Arrest
PPG: Photoplethysmography
PKP2: Plakophilin-2
PLN: Phospholamban
QT: QT Interval
RAF: Resuscitation Academy Foundation
ROC: Receiver Operating Characteristic
RRV: Respiratory Rate Variability
SCA: Sudden Cardiac Arrest
SCD: Sudden Cardiac Death
SCD-HEFT: Sudden Cardiac Death in Heart Failure Trial
SCN5A: Sodium Voltage-Gated Channel Alpha Subunit 5 Gene
SIDS: Sudden Infant Death Syndrome
SQTS: Short QT Syndrome
STEMI: ST-Elevation Myocardial Infarction
SVT: Supraventricular Tachycardia
VA: Ventricular Arrhythmia
VFRisk: Ventricular Fibrillation Risk Score
VF: Ventricular Fibrillation
VT: Ventricular Tachycardia
WPW: Wolff-Parkinson-White Syndrome
